# Development and Performance Evaluation of a Low-Cost Portable PM_2.5_ Monitor for Mobile Deployment

**DOI:** 10.3390/s22072767

**Published:** 2022-04-04

**Authors:** Mingjian Chen, Weichang Yuan, Chang Cao, Colby Buehler, Drew R. Gentner, Xuhui Lee

**Affiliations:** 1 Yale-NUIST Center on Atmospheric Environment, Nanjing University of Information Science and Technology, Nanjing 210044, China; 20191207006@nuist.edu.cn (M.C.); chang.cao@nuist.edu.cn (C.C.); 2 Jiangsu Key Laboratory of Agriculture Meteorology, Nanjing University of Information Science and Technology, Nanjing 210044, China; 3 School of the Environment, Yale University, New Haven, CT 06511, USA; xuhui.lee@yale.edu; 4 Department of Chemical & Environmental Engineering, School of Engineering and Applied Science, Yale University, New Haven, CT 06511, USA; colby.buehler@yale.edu (C.B.); drew.gentner@yale.edu (D.R.G.); 5 Solutions for Energy, Air, Climate and Health (SEARCH), School of the Environment, Yale University, New Haven, CT 06511, USA

**Keywords:** low-cost sensor, air quality, hotspot, public health, portable monitor, crowdsourcing

## Abstract

The concentration of fine particulate matter (PM_2.5_) is known to vary spatially across a city landscape. Current networks of regulatory air quality monitoring are too sparse to capture these intra-city variations. In this study, we developed a low-cost (60 USD) portable PM_2.5_ monitor called Smart-P, for use on bicycles, with the goal of mapping street-level variations in PM_2.5_ concentration. The Smart-P is compact in size (85 × 85 × 42 mm) and light in weight (147 g). Data communication and geolocation are achieved with the cyclist’s smartphone with the help of a user-friendly app. Good agreement was observed between the Smart-P monitors and a regulatory-grade monitor (mean bias error: −3.0 to 1.5 μg m^−3^ for the four monitors tested) in ambient conditions with relative humidity ranging from 38 to 100%. Monitor performance decreased in humidity > 70% condition. The measurement precision, represented as coefficient of variation, was 6 to 9% in stationary mode and 6% in biking mode across the four tested monitors. Street tests in a city with low background PM_2.5_ concentrations (8 to 9 μg m^−3^) and in two cities with high background concentrations (41 to 74 μg m^−3^) showed that the Smart-P was capable of observing local emission hotspots and that its measurement was not sensitive to bicycle speed. The low-cost and user-friendly nature are two features that make the Smart-P a good choice for empowering citizen scientists to participate in local air quality monitoring.

## 1. Introduction

Outdoor air pollution is estimated to cause over three million premature deaths per year worldwide, and this number is projected to double by 2050 under a business-as-usual emission scenario [1]. A prominent air pollutant is fine particulate matter with a diameter less than or equal to 2.5 μm (PM_2.5_). Based on a large body of scientific evidence accumulated in the past decades, the U.S. Environmental Protection Agency (EPA) has concluded a causal relationship between PM_2.5_ exposure (both short-term and long-term) and mortality [2]. Adverse health effects associated with PM_2.5_ exposure include, but are not limited to, respiratory and cardiovascular morbidity, cancer risks, and possible harm to the nervous system [2,3,4].

Fixed site monitor (FSM) networks are important tools to monitor ambient air quality, including PM_2.5_ concentrations, for regulatory purposes [5,6]. Regulatory FSMs have high capital and maintenance costs, and they are sparsely distributed. For example, in the U.S., on average there are only 2 to 5 regulatory FSMs per 1 million people and in 1000 km^2^ with continuous regulatory monitoring [7]. Despite the high accuracy and reliability of regulatory FSMs, they only represent point estimates of ambient air quality with limited temporal resolution (typically hourly). However, PM_2.5_ concentration typically varies spatially over short intra-city scales due to variations in land use and source distributions, referred to as spatiotemporal variability [7,8].

Some current air quality management efforts focus on improving spatial and temporal resolution beyond regulatory FSMs, and the paradigm of air pollution monitoring is shifting towards applications of low-cost air sensors [9]. Although it is recognized that low-cost air sensors will likely not meet stringent requirements for regulatory monitoring purposes, these sensors can be useful in many nonregulatory supplemental and informational monitoring (NSIM) applications, such as understanding local air quality trends, identifying emission hotspots, supplemental monitoring, and promoting educational/environmental awareness [9,10]. The cost of these sensors typically falls in the range of USD 100 to 2500, representing a substantial reduction from the cost of regulatory FSMs [11]. Low-cost air sensors can be deployed in large quantities, and they often report data at intervals of minutes and even seconds [9,12]. The existing low-cost air sensor networks have spatial coverage ranging from regional to international, such as the Real-Time Multi-Pollutant Sensors (RAMP) in Pittsburgh, PA, USA [13], the Solutions for Energy, Air, Climate and Health (SEARCH) network in Baltimore, MA, USA [14], AirCasting [15], and Air Quality Egg [16] mostly in the U.S. and Europe, hackAIR mostly in Europe [17], PurpleAir mostly in North America and Europe [18], and others [19]. More sensor nodes help improve the spatial resolution of these sensor networks, and in the near future, low-cost sensor networks offer the potential to monitor ambient PM_2.5_ concentration at the neighborhood scale.

In addition to stationary low-cost sensor networks, mobile sensors can be used to characterize intra-city variations in air quality. Mobile monitoring platforms, such as those based on cars and bicycles, can further improve the spatial and temporal resolution of PM_2.5_ monitoring by providing real-time snapshots of PM_2.5_ concentration profiles along chosen paths. Some mobile systems consist of research-grade monitors mounted in special vehicle-based laboratories [7,20,21,22,23]. Others are based on commercial, portable monitors mounted on bicycles [24,25,26,27,28,29]. However, commercially available portable monitors are relatively expensive (typically in the order of thousands of USD), and it is not feasible for deployment in large quantities.

To quantify intra-city spatiotemporal PM_2.5_ concentration variations with mobile platforms, repetitive measurements over the same routes are recommended [7,15]. Such measurements require a serious time commitment that researchers and air quality specialists may not be able to afford and are costly with commercial monitors. In this regard, low-cost sensors are advantageous because they can be deployed in large quantities. Distributing low-cost sensors to citizens for monitoring of air quality may contribute to citizen science [11]. Involving citizen scientists in air quality monitoring has been referred to as participatory monitoring [9]. Such collective citizen efforts are also called crowdsourcing [30]. A well-known crowdsourcing practice involves citizen participation in science projects through the use of smartphones [31]. Low-cost PM_2.5_ sensors offer an opportunity for citizen scientists concerned with local air quality to participate in data collection, thus expanding the spatial and temporal coverage of mobile monitoring.

This study aimed (1) to develop a low-cost portable PM monitor, (2) to evaluate its performance against a research-grade instrument, and (3) to demonstrate its potential for mobile deployment with actual street transect measurements. This study is part of a project called “Biking for Science and Health”, whose goal is to develop smart environmental sensors for use on bicycles to monitor heat stress and air quality in urban environments.

## 2. Materials and Methods

### 2.1. Smart-P Development

**Figure 1:**
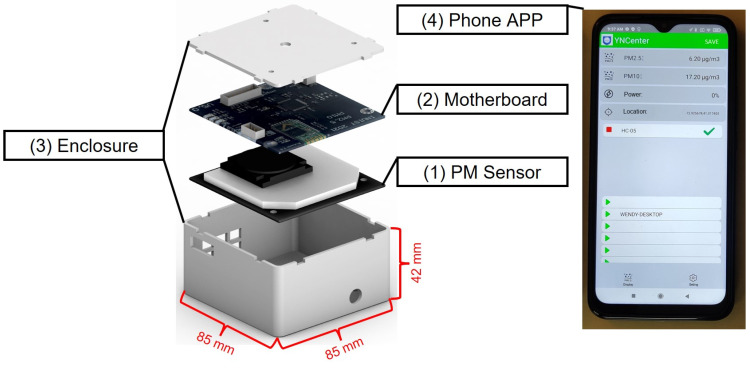
The four major modules of a Smart-P monitor.

In the following, the term “monitor” refers to an entire measuring system consisting of a sensor, signal processing, data transmission and storage modules, a power supply, and an enclosure. The low-cost portable PM monitor is called Smart-P. Its design principle is similar to that deployed for Smart-T, a low-cost portable monitor for temperature and humidity for use on bicycles [32]. The key attributes of these monitors are lightweight, low cost, low power consumption, ability to communicate and geolocate the data (via the cyclist’s smartphone), and potential to be deployed in large quantities. Figure 1 is an illustration of the four major modules of a Smart-P monitor, including (1) an off-the-shelf PM sensor; (2) a motherboard; (3) an enclosure; and (4) a smartphone application (app) called YNCenter. The total cost of the hardware (i.e., modules 1 to 3) is around USD 60 per monitor (see Table S1 for the cost breakdown), which is lower than the lower bound (i.e., USD 100) of the typical definition of low-cost air monitors [11].

Several other researcher groups have developed low-cost PM monitors that can be used in mobile monitoring [33,34,35,36,37,38,39,40,41]. The Smart-P monitor was inspired in part by their work and represents an effort to overcome operational challenges by substantially reducing the weight, size, and cost.

#### 2.1.1. PM Sensor

The sensor module of Smart-P is an off-the-shelf SDS011 PM sensor (hereafter referred to as SDS011) manufactured by Nova Fitness Co., Ltd. (Jinan, China) [42]. The SDS011 is based on the principle of light scattering to measure the concentration of particles with aerodynamic diameters from 0.3 to 10 μm in the air. A built-in fan draws air into a chamber equipped with a laser diode, where PM_2.5_ and PM_10_ particle concentrations are measured. In this paper, we focused our evaluation exclusively on its PM_2.5_ performance. The SDS011 sensor specifications meet several criteria needed for mobile monitoring of PM_2.5_ concentration in an urban environment. For example, its short corresponding time of 1 s contributes to collecting data in high spatial and temporal resolutions. Its wide detection range of 0.0 to 999.9 μg m^−3^ also allows measurement of low levels as well as hotspots of PM_2.5_ concentration.

The data quality of the SDS011 is well-documented in the literature, primarily based on stationary measurements. For example, Liu et al. conducted collocation measurements with three SDS011 sensors at a regulatory air monitoring station in Oslo, Norway for a four-month period [43]. Their collocation results indicated a strong correlation (*r* > 0.97) between the three SDS011 sensors and good linearity against a regulatory PM_2.5_ monitor (*R*^2^ from 0.55 to 0.71). On an hourly average basis, the three tested sensors had accuracy ranging from 81 to 98% compared with the reference instrument. The accuracy decreased substantially with high relative humidity of more than 80%. Genikomsakis et al. compared an SDS011 sensor to a commercial high-quality optical particle sizing instrument (Model: TSI OPS 3330), which had a U.S. National Institute of Standards and Technology (NIST) traceable calibration [34]. The SDS011 sensor reading was highly correlated to the TSI OPS 3330 reading, with an *R*^2^ of 0.933 with one-minute average data for a three-day measurement period. Badura et al. simultaneously evaluated four models of off-the-shelf low-cost PM_2.5_ sensors in a half-year period, including three monitors of each of the following models: SDS011, Winsen ZH03A (Henan, China), Plantower PMS7003 (Beijing, China), and Alphasense OPC-N2 (Essex, UK). Among the four models, the SDS011 demonstrated the best precision (1-min average data) and the second-best linearity against a reference regulatory-grade PM_2.5_ monitor [44]. These studies did not investigate the precision performance of the SDS011 on mobile platforms.

Successful use of the SDC011 on bicycles requires solutions to several logistical challenges. In the applications cited above, the SDS011 sensor was used at a fixed location and usually with easy access to A/C power. The sensor readout was achieved via a USB port connection to a computer. Such power and data arrangements would not be feasible for mobile deployment on a bicycle. Potential adverse impacts on data quality may arise due to biking speed [10,11]. In addition, easy installation and handling are desirable to promote the participation of citizen scientists. In the following, we briefly describe how we overcame these challenges.

#### 2.1.2. Motherboard

**Figure 2:**
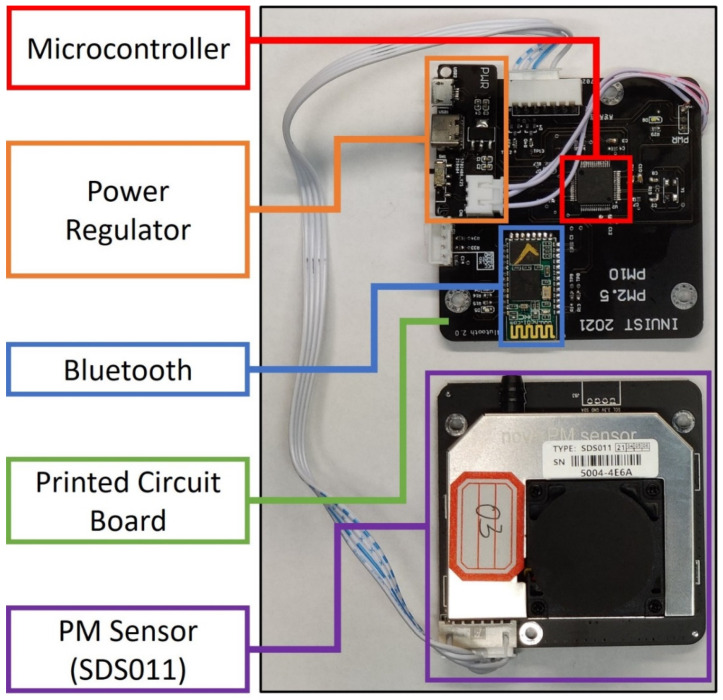
Internal view of a Smart-P monitor.

We integrated the electronic components onto a specialized motherboard, as shown in Figure 2. The motherboard is a printed circuit board (PCB) with a microcontroller, a power regulator, and a Bluetooth module. The motherboard reduces the need for wires and, thus, minimizes the chance of loose connections or short circuits during mobile measurements where persistent vibrations occur. Additional benefits of the motherboard include ease of mass production, size reduction, and uniformity of electrical characteristics among multiple monitors. The microcontroller (part number: STM32F401RBT6) is programmed to transmit data reported by the SDS011 to the YNCenter app via Bluetooth (part number: HC-05). It accepts commands, such as setting sampling intervals, from the YNCenter app (via Bluetooth) and sends the commands to the SDS011. The power regulator (part number: RT8096CHGJ5) allows Smart-P to use 5V input via a USB-C connection. The power source, supplied by the user, can be a power bank and a smartphone charger for mobile and stationary usage, respectively.

When sampling at 1 Hz, Smart-P has a rated current of 105 mA, corresponding to a power rating of 0.515 W, about 70% of which is consumed by the SDS011 sensor. At this sample rate, a 10,000 mAh power bank, which weighs about 210 g, lasts 95 h. Extended battery life can be achieved by sampling less frequently and putting the SDS011 sensor into sleep mode between sampling intervals. The sleep current is less than 4 mA based on the SDS011 specifications.

#### 2.1.3. Enclosure

We designed a 3D-printed enclosure to protect the electronics and the sensor from physical damage and to reduce vibrations during mobile measurements. It houses the electronics and the sensor while allowing air to flow in and out. The enclosure has a dimension of 85 × 85 × 42 mm. The total weight of a Smart-P monitor, including the enclosure, the electronics, and the sensor, is 147 g, comparable to the mass of a typical smartphone.

According to the manufacturer, connecting the SDS011 to an inlet tube is optional. If an inlet sampling tube is desired, it recommends that the tube be less than 1 m in length. In our tests, we found that tubes of 1 m in length (Teflon, inside diameter 6 mm, outside diameter 8 mm) reduced the sensor reading to near-zero values regardless of ambient PM levels, presumably due to particle deposition onto the tube walls, including by electrostatic losses. Our final choice for the Smart-P was a Teflon tube with a length of 5 cm. This inlet tube is long enough to ensure that the SDS011 sensor can draw air from outside of the enclosure and is short to reduce particle deposition. Another benefit of a short tube is decreased travel time of the sampled air and therefore a fast response in mobile measurements.

#### 2.1.4. Smartphone App

We developed the YNCenter app for data displaying, time recording, position geolocating, and data storage in real-time using a smartphone. The app turns the user’s phone into a data logging and geolocating device. Its user interface is shown in Figure 1. Once connected with a Smart-P monitor, the app displays the real-time PM_2.5_ and PM_10_ concentrations. For each data log, the app records local date and time, GPS coordinates (latitude and longitude), and PM_2.5_ and PM_10_ concentrations. The app allows the user to customize the data logging interval from 1 Hz for high-resolution mapping to a long interval (e.g., 30 s) to conserve battery or at times of stable PM concentration. The app utilizes the built-in real-time clock and the GPS module of the smartphone to obtain the date, time, and location data. This arrangement reduces the cost of Smart-P. Data are stored locally in the paired smartphone as comma-separated values (CSV) files, with file names representing the exact end date and time (in the format “YYYYMMDDHHMMSS”) of the mobile measurements. Users can inspect the saved data by exporting them to a computer and visualizing them. Privacy-sensitive information, such as GPS coordinates at the start and end of a street transect measurement, can be removed manually before sharing the data or depositing it to a public archive.

The app, in its beta version, was only available for Android devices at this time. An app with the same functionality will be available for the IOS operating system in the near future.

### 2.2. Smart-P Operation

**Figure 3:**
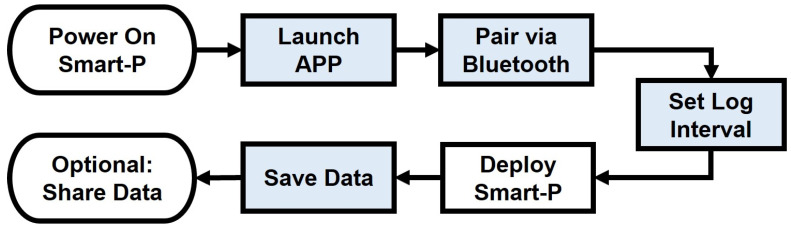
Step-by-step operation of Smart-P for measuring PM_2.5_ concentrations. Shaded boxes indicate app operations.

The step-by-step standard operating procedure of Smart-P is illustrated in Figure 3. Once Smart-P is powered up, most operations are achieved via the YNCenter app. The app was designed to be intuitive and user-friendly from the perspective of citizen scientists without special training in air quality monitoring. When deployed in large quantities, Smart-P has the potential to contribute to crowdsource-based research projects.

### 2.3. Field Comparison with a Regulatory-Grade PM_2.5_ Monitor

**Figure 4:**
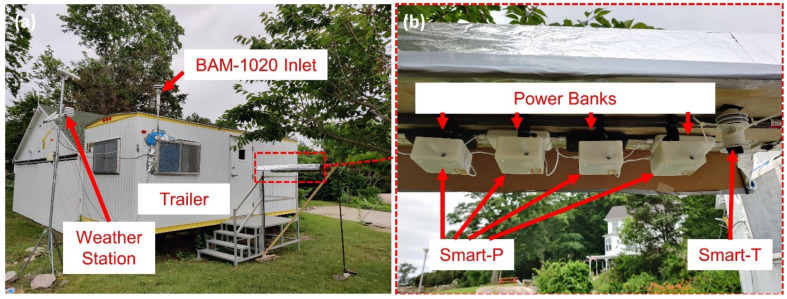
Collocation at Yale Costal Field Station (YCFS): (**a**) YCFS site; (**b**) Smart-P installation.

Collocating portable monitors with stationary reference air monitors prior to mobile deployment is a common practice in the field of using portable monitors for air quality monitoring [45,46,47,48,49,50,51,52,53,54]. Collocation refers to operating sensors and a reference instrument at the same time and the same place under real world conditions [10]. To evaluate the Smart-P performance, we collocated four monitors (IDs: 501, 502, 503, and 504) at the Yale Costal Field Station (YCFS) in Guildford, Connecticut, U.S. (41.2583° N, 72.7312° W), shown in Figure 4 with site description and characterization in prior work [21,55]. The YCFS (Figure 4a) is equipped with a Met One BAM-1020 monitor to measure ambient PM_2.5_ with hourly resolution. The BAM-1020, based on beta ray attenuation, is a U.S. regulatory-grade federal equivalent method [56]. The BAM-1020 has a detection limit of less than 4.8 μg m^−3^ and a range of 0–1000 μg m^−3^ [21]. The stainless-steel inlet to the BAM-1020 monitor was positioned on a small tower at a height of 3 m above the ground to sample ambient air, as shown in Figure 4a. The BAM-1020 monitor was situated inside a trailer. Hourly meteorological conditions, including temperature and relative humidity, were reported by an adjacent weather station.

We installed four Smart-P monitors beside the trailer under a rack, as shown in Figure 4b. The rack protected the Smart-P monitors, mounted at a height of about 2 m above ground, from rain and sunlight exposure, and allowed uninterrupted airflow from all other directions. Each Smart-P monitor was connected to a 20,000 mAh power bank to ensure a continuous power supply for at least three days. During the measurement campaign, we performed daily instrument checks, data retrieval, and replaced the power banks as needed. Each Smart-P monitor was paired with a RedMi 9 smartphone with 4 GB memory for monitoring and data storage.

The collocation measurements took place in July 2021. Measurements were made intermittently due to summer storms. In total, six measurements were conducted, with each measurement lasting 7 to 52 h. The Smart-P monitors recorded data every 30 s; these were averaged to hourly values for comparison with the measurement made with the BAM-1020 monitor.

For the six measurements at YCFS, we collected 17,891 to 19,664 readings, covering 152 to 163 valid hours (see Tables S2 and S3 for details). A valid hour refers to an hour with more than 89 readings. The number of valid readings varied among the Smart-P monitors due to occasional data communication issues. The manufacturer-recommended working conditions for SDS011 sensors are temperatures from −10 to 50 °C and relative humidity (RH) below 70%. However, because of the nature of ambient measurements, meteorological conditions were not controlled to fully satisfy these recommendations. The temperature during the measurements (13–30 °C) was within specifications and RH (38–100%) was beyond the recommended range at this coastal site in the summertime.

We used linear regression to quantify bias and linearity between two monitors. Biases were evaluated with the slope and intercept of the regression. Linearity was evaluated with the coefficient of determination (*R*^2^). Other error statistics included the root mean square error (RMSE), the mean absolute error (MAE), and the mean bias error (MBE).

### 2.4. Additional Tests

We conducted three types of tests to prepare the Smart-P for real-world deployments. In all these tests, the temperature and RH fell within the manufacturer-recommended working conditions for the SDS011. The first type was indoor tests with stationary monitoring. Four Smart-P monitors (IDs: 501, 502, 503, and 504) were placed side-by-side on a desk in an apartment. One goal of the indoor tests was to evaluate whether the Smart-P could capture PM_2.5_ emission episodes, which were generated by two cooking events with an electric range burner. In the first cooking event, about 50 mL of vegetable oil was heated to generate noticeable smoke. The second cooking event was boiling water and only a small amount of smoke was generated from the residual oil left on the burner surface from the first cooking event. These two cooking events generated PM_2.5_ episodes with drastically different magnitudes. They lasted about five minutes. Data were logged at 2 s intervals.

The second type of test was mobile measurements using a passenger car in New Haven, CT, USA, and Hangzhou, Zhejiang, China. In both measurements, data were logged at 1 Hz and the driving speed varied from 0 to 90 km h^−1^. The New Haven test took place on 15 June 2021, from 4:02 PM to 4:52 PM. Four Smart-P monitors (IDs: 501, 502, 503, and 504) were mounted on the front windshield of a passenger car, with the inlets facing forward. We measured a 15 km street transect across major parts of Yale University, its adjacent residential neighborhoods (East Rock and Dixwell), and downtown New Haven. The transect mainly consisted of one- and two-lane arterial streets. At the time of the test, the nearest regulatory air monitor (41.301157° N, 72.902887° W) reported an hourly average PM_2.5_ concentration of 7.5 μg m^−3^.

The Hangzhou car test took place on 17 April 2021, from 9:40 AM to 12:40 PM. One Smart-P monitor (ID: 512) was mounted on the roof rack of a passenger car with the inlet facing forward. We measured a 30 km transect across three major urban districts in Hangzhou, including Shangcheng, Gongshu, and Xihu. The transect, consisting of multi-lane arterial roads and elevated expressways, crossed a variety of urban landscapes, such as high-rise residential buildings, high-rise office buildings, recreational parks, industrial areas, and construction zones. At the time of the test, the ambient hourly-average PM_2.5_ at the nearest regulatory monitoring station (30.253103° N, 120.212997° E) was 74 μg m^−3^.

The third type of test was also carried out in outdoor conditions, with the Smart-P mounted on a bicycle. The bicycle measurements were conducted in New Haven and in Nanjing, Jiangsu, China. In both measurements, data were logged at 1 Hz and the cycling speed varied from 0 to 23 km h^−1^. The New Haven bicycle test took place on 4 August 2021, from 11:00 AM to 11:30 AM. We mounted four Smart-P monitors (IDs: 501, 502, 503, and 504) on the rear rack of a bicycle with two inlets facing left and two facing right to reduce the effect of flow blockage by the cyclist. We measured a 5 km transect around the Yale University main campus. At the time of the test, the nearest regulatory air monitor (41.301157° N, 72.902887° W) reported an hourly average PM_2.5_ concentration of 8.5 μg m^−3^.

The Nanjing bicycle test took place on 14 July 2021, from 5:24 PM to 6:00 PM. We mounted a Smart-P monitor (ID: 508) on the handlebar. We measured a 6 km transect centered at the Nanjing Olympic Sports Stadium. At the time of the test, the nearest regulatory air monitor (32.197971° N, 118.719017° E) reported an hourly average PM_2.5_ concentration of 41 μg m^−3^.

For these additional tests, a reference monitor was not placed next to the Smart-P monitors. This was because reference monitors for PM_2.5_, including U.S. EPA federal reference and equivalent methods, are primarily designed for stationary use at designated sites. As an example, the Met One BAM-1020 monitor operates at 100 to 230 volts alternating current and is 310 mm high × 430 mm wide × 400 mm deep. The BAM-1020 was designed to mount in a temperature-controlled enclosure. The sampling inlet was designed to mount through the roof of the enclosure. Hence, it is logistically challenging to operate it on a mobile platform, such as a passenger car or a bicycle. Moreover, reference monitors typically report data on an hourly basis, as opposed to the up to second-by-second basis of many low-cost sensors, including the SDS011 sensor in the Smart-P. Therefore, side-by-side comparison to a reference monitor was not conducted in these additional tests. Nevertheless, to indicate whether the Smart-P readings were reasonable, hourly average PM_2.5_ concentration data were obtained from the nearest regulatory monitor wherever possible.

### 2.5. Precision

For the tests made simultaneously with multiple Smart-P monitors, we quantified the precision, or unit-to-unit variance, among the monitors using standard deviation (SD) and coefficient of variation (CV) [44]. We first calculated the 1 min mean for each sensor and then calculated the SD and CV using these 1 min means. After that, we averaged the 1 min SD and CV to obtain the mean SD and CV for the whole test. We also quantified SD and CV on an hourly basis using the data collected at YCFS. We used a 10% CV as a threshold for evaluating precision performance [57,58,59].

## 3. Results

### 3.1. Performance Evaluation against BAM-1020

**Figure 5:**
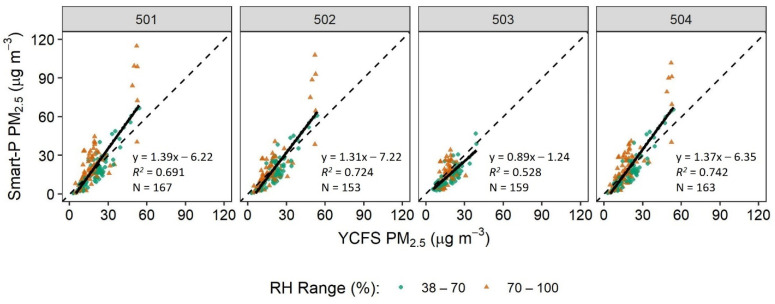
Linear regression (black solid lines) of hourly PM_2.5_ concentration of each of the four Smart-P monitors (IDs: 501, 502, 503, and 504) versus the BAM-1020 monitor at the Yale Coastal Field Station (YCFS). Dotted lines are diagonal (1:1) lines.

Figure 5 shows the comparison of hourly PM_2.5_ concentration between each of the four Smart-P monitors and the BAM-1020 monitor at the YCFS. Under the full range of measured RH, the Smart-P monitors showed some biases against the BAM-1020, as indicated by the regression slopes and intercepts. These biases are in part attributable to the differences in physical and chemical properties between the particles used in manufacturer calibration of the SDS011 and real-world ambient particles [44]. An additional source of bias is associated with varying RH, which affects particle light scattering properties due to water uptakes, and has been shown to be important for low-cost PM measurements based on light scattering [60,61].

All four Smart-P monitors demonstrated moderate to high linearity against the BAM-1020, as indicated by *R*^2^ of 0.528 to 0.742 (Table 1). A lower *R*^2^ for Smart-P 503 than the other three monitors was attributed to the narrower range of measured PM_2.5_ concentration within its dataset, and *R*^2^ values typically decrease with lower measured PM_2.5_ concentration range for low-cost PM_2.5_ sensors, including the SDS011 [44].

Light scattering PM_2.5_ sensors are subject to interferences from variations in RH [10,45,61]. When filtering for data points with RH > 70%, the results verified that operating Smart-P in RH ≤ 70% conditions reduced biases, improved the linearity, and reduced RMSE and MAE against the BAM-1020 (Table 1).

Smart-P errors occurred in both directions. For Smart-P monitors 501, 502, and 504, for which a wider range of PM_2.5_ concentration was collected, the MBEs of RH ranges 38–70 and 70–100 were consistently negative and positive, respectively. These results indicated that operating Smart-P in low (≤70%) and high (>70%) RH environment tends to underestimate and overestimate PM_2.5_ concentration, respectively. Across the entire measured RH range from 38 to 100%, these four Smart-P monitors had MBEs from −3.0 μg m^−3^ to 1.5 μg m^−3^. The relative biases, that is, the ratio of the MBE to the mean concentration measured by BAM-1020, were 8, −5, −19, and 4% for Smart-P 501, 502, 503, and 504, respectively, based on the entire measured RH range (Table 1).

The Smart-P seemed to perform reasonably well in RH > 70% conditions. Based on data collected in this high humidity range, Smart-P maintained high linearity, with *R*^2^ > 0.68 for Smart-P monitors 501, 502, and 504, which covered relatively wide ranges of hourly PM_2.5_ concentrations. For the four Smart-P monitors, the MBE varied between −0.9 and 5.1 μg m^−3^ and the relative bias was between −6 and 27% in RH > 70% conditions (Table 1).

For the overlapping hours during the collocated measurements among the four Smart-P monitors, satisfactory precision was achieved, as indicated by the SD and CV summarized in Table 2. Similar to the bias performance statistics, the precision was moderately sensitive to RH. Operating the Smart-P with RH ≤ 70% led to an SD of 0.7 to 1.0 μg m^−3^ and CV of 6%. The precision was adequate in view of the range of PM_2.5_ concentration (3 to 54 μg m^−3^ based on BAM-1020). The SD and CV were higher under high humidity conditions (RH > 70%), in the range of 2.2 to 2.5 μg m^−3^ and 10 to 12%, respectively, but calibrations to correct for RH could reduce the effects [61]. For the entire measured range of RH values, the SD and CV were 1.6 μg m^−3^ and 9%, respectively, based on the comparison to each other among the four monitors (Table S4). The high precision was also indicated by *R*^2^ of 0.94 to 0.99, shown in the parity plots of each monitor versus the mean of the remaining monitors (Figures S1 and S2).

### 3.2. Response to Emission Episodes in Stationary Measurements

**Figure 6:**
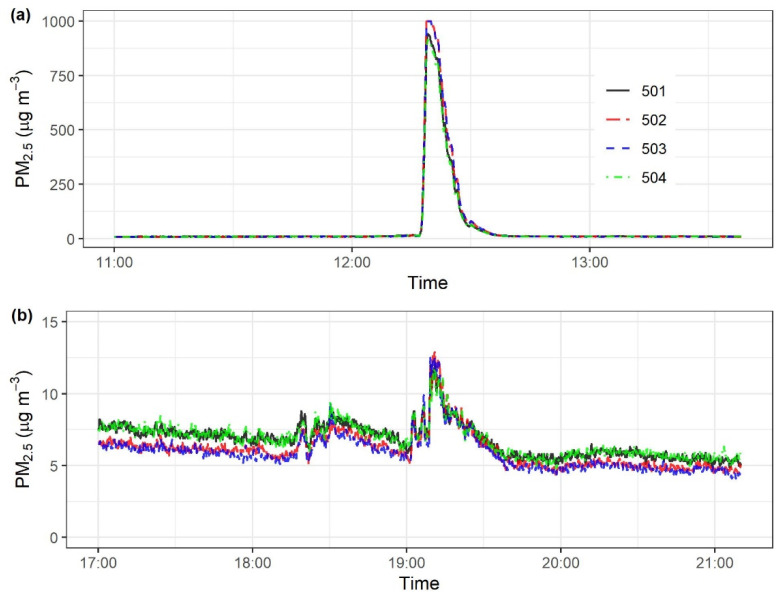
Variations in indoor 1 Hz PM_2.5_ concentrations measured by four Smart-P monitors (IDs: 501, 502, 503, and 504) during two simulated cooking events: (**a**) with oil and (**b**) with water.

Figure 6 shows the variations in indoor PM_2.5_ concentrations during the two cooking events. All four Smart-P monitors responded quickly and consistently to PM_2.5_ emission episodes. During the first episode, they captured the high PM_2.5_ peak started at 12:17, about one minute after the cooking stove was turned on, with peak magnitude close to the upper bound of the SDS011 detection range (i.e., 999.9 μg m^−3^). During the second event, they captured a smaller PM_2.5_ peak of 12 to 13 μg m^−3^ at about the same time (19:10). The two cooking events verified that Smart-P can measure short emission episodes of both high and low emission intensities. The variations among the monitors were within the rated relative error by the manufacturer, i.e., ±15%. For both cooking events, the readings were highly precise, as indicated by *R*^2^ of 1 for the noon high emission episode (Figure S3) and *R*^2^ of 0.93 to 0.95 for the evening low emission episode (Figure S4) on a 1 min average basis. The SD and CV of the test-average concentrations among the four monitors were 3.8 μg m^−3^ and 6% for the high emission episode and 0.5 μg m^−3^ and 8% for the low emission episode, respectively (Table S4).

### 3.3. Mobile Measurements along Street Transects

**Figure 7:**
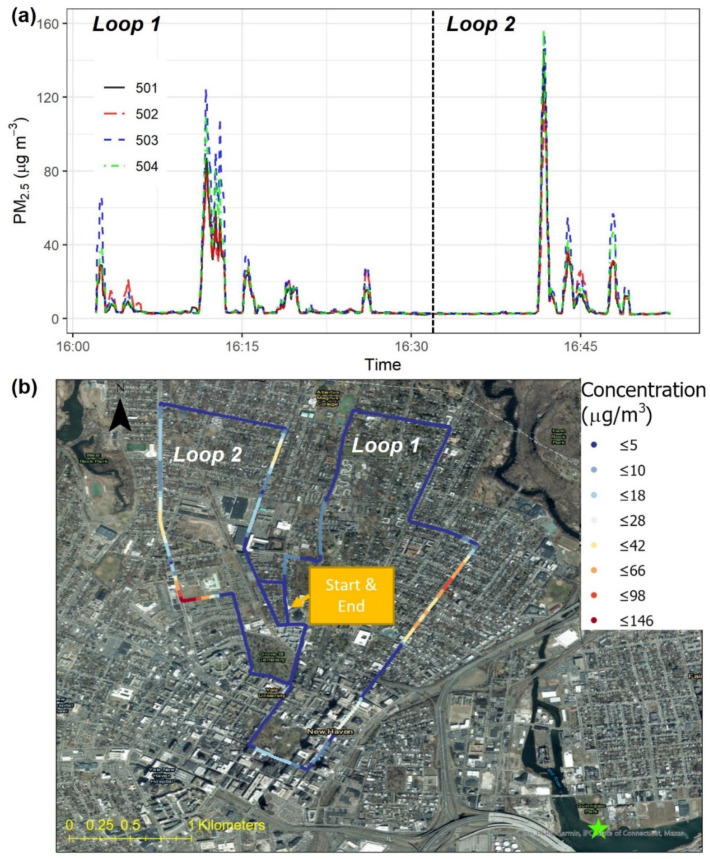
Car-based test in New Haven, Connecticut, U.S.: (**a**) time series of PM_2.5_ concentrations measured by four Smart-P monitors (IDs: 501, 502, 503, and 504) and (**b**) map visualization of PM_2.5_ concentrations measured by one Smart-P monitor (IDs: 501). The driving direction in both loops was clockwise. At the time of this test, the hourly average PM_2.5_ concentration from the nearest regulatory monitor (green star) was 7.5 μg m^−3^.

Figure 7a shows the time series of PM_2.5_ concentrations measured by the four Smart-P monitors during the car-based test in New Haven. The four monitors recorded similar concentrations ranging from 2 to 164 μg m^−3^, with a test-wide average of 9 to 12 μg m^−3^. This range of intra-city variations encompassed the corresponding hourly PM_2.5_ concentration from the EPA monitor (7.5 μg m^−3^). Approximately 60% of the Smart-P readings were below 7.5 μg m^−3^, with a mean of 3 to 4 μg m^−3^, depending on the Smart-P monitor. The remaining Smart-P readings were above 7.5 μg m^−3^, with a mean of 25 to 35 μg m^−3^, depending on the Smart-P monitor. These Smart-P readings indicated that the regulatory monitor was not representative of the spatial and temporal variations in ambient PM_2.5_ concentration within the city area.

Figure 7b shows a map visualization of the spatially resolved PM_2.5_ concentrations measured with Smart-P 501. The other three Smart-P monitors (i.e., 502, 503, and 504) showed similar spatial patterns (Figure S5). All four monitors showed sharp changes at several locations. The first peak at 16:02 occurred at the start of the measurement when the car was backed out of a parking spot. At this time, the Smart-P monitors sampled tailpipe emissions during the cold start phase of the car engine. Similarly, the subsequent PM_2.5_ peak with magnitudes between 10 and 20 μg m^−3^ from 16:03 to 16:04 was likely attributed to the car stopping in front of a stop sign when the car engine was still in the cold start phase, and a cold start contributes the most (>50%) PM emissions in a given driving cycle of gasoline vehicles [62]. The following peaks may include potential contributions from traffic-related sources. For example, the high concentrations recorded on Orange Street (Loop 1) between 16:11 and 16:14 occurred near dense traffic, including a diesel bus moving 10 to 20 m ahead of the measurement car. The PM_2.5_ concentration peaks at the intersection of Dixwell Avenue and Foote Street (Loop 2) at about 16:41 was potentially contributed to by an idling diesel garbage truck, as diesel engines are known emitters of PM_2.5_ [63,64]. However, these peaks could also be linked to cooking emissions from nearby restaurants, and restaurants can emit high PM_2.5_ [8].

The four monitors were all able to detect these highly localized patterns, indicating high precision. On a 1 min average basis, the four Smart-P monitors had a precision of 1.8 μg m^−3^ (one SD) and 13% based on the CV (Table S4). The relatively high CV for the car test was caused by concentrated plumes, which were not well-mixed. If the first five minutes of the observation were excluded, the precision improved to 1.4 μg m^−3^ and 10%. The *R*^2^ among the monitors was in the range of 0.97 to 0.99 (Figure S6).

**Figure 8:**
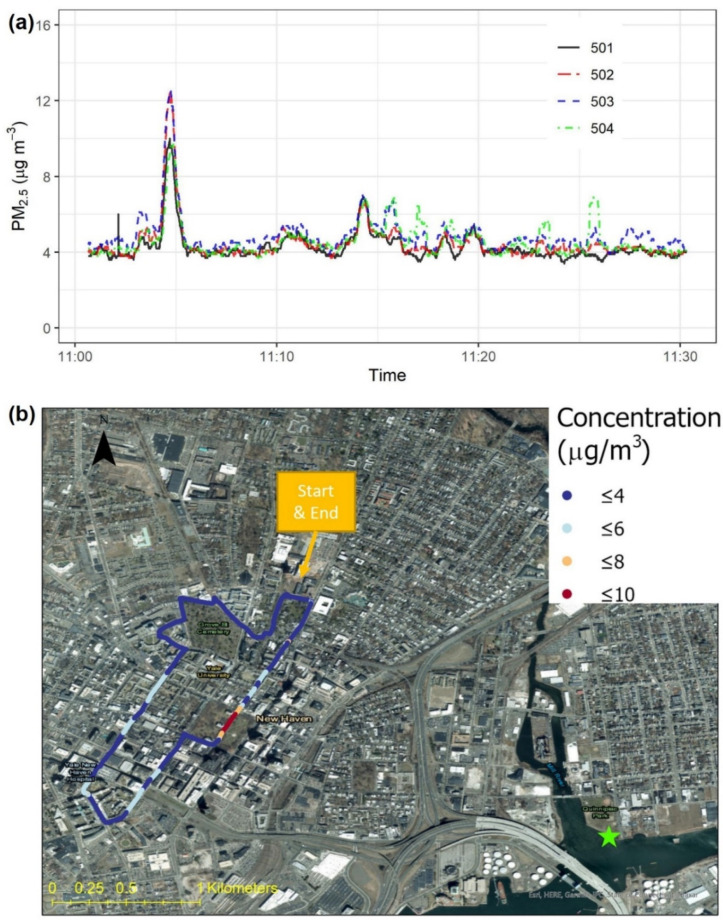
Bicycle-based test in New Haven, Connecticut, U.S.: (**a**) time series of PM_2.5_ concentrations measured by four Smart-P monitors (IDs: 501, 502, 503, and 504) and (**b**) map visualization of PM_2.5_ concentrations measured by one Smart-P monitor (IDs: 501). The biking direction was clockwise. At the time of this test, the hourly average PM_2.5_ concentration from the nearest regulatory monitor (green star) was 8.5 μg m^−3^.

Figure 8 shows intra-city variations in PM_2.5_ concentration, measured with the same four Smart-P monitors mounted on a bicycle, in New Haven. This transect consisted of narrow streets, sidewalks, and greenways, some of which were not accessible by cars. The concentration was lower than that shown in Figure 7, in part because the cyclist deliberately avoided vehicle traffic most of the time. The concentration was in the range of 3 to 13 μg m^−3^. In comparison, the hourly average concentration from the closest regulatory monitor corresponding to the time of our bicycle measurement was 8.5 μg m^−3^. A small PM_2.5_ peak of 10 to 13 μg m^−3^ was recorded at the New Haven Green at about 11:04, near an idling bus at a bus stop there. This hotspot was detected by all four monitors (Figure S7). The precision (one SD) was 0.3 μg m^−3^ and 6% (CV) based on 1 min average PM_2.5_ concentrations (Table S4).

**Figure 9:**
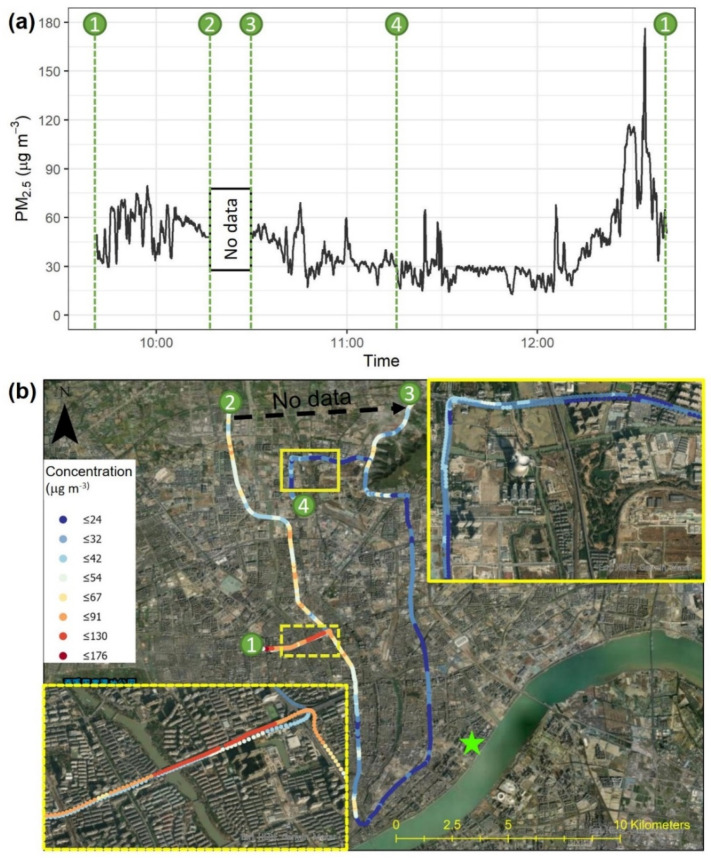
Car-based test in Hangzhou, Zhejiang, China using one Smart-P monitor (ID: 512): (**a**) time series of PM_2.5_ concentrations and (**b**) map visualization of PM_2.5_ concentrations. At the time of the test, the hourly average PM_2.5_ concentration from the nearest regulatory monitor (green star) was 74 μg m^−3^.

The car-based test in Hangzhou (Figure 9) demonstrated the performance of Smart-P in an environment with higher background PM_2.5_ concentrations than in New Haven. The car started at point 1 in an urban–rural fringe area, moved north to point 2, and then to point 3 and point 4. After that, it turned around, got on an expressway, traveled on the expressway loop for 18 km, and then returned to the starting point 1. The road near point 1 was shared by many heavy-duty trucks, and there was roadside construction. On the return trip to point 1, the monitor recorded a peak concentration of 176 μg m^−3^. Some variations in PM_2.5_ concentration were observed on the expressway, which may have been influenced by changes in traffic volume. During the whole transect, the concentration varied in the range of 13 to 176 μg m^−3^. For comparison, the hourly PM_2.5_ concentration at the nearby regulatory monitoring site was 74 μg m^−3^.

**Figure 10:**
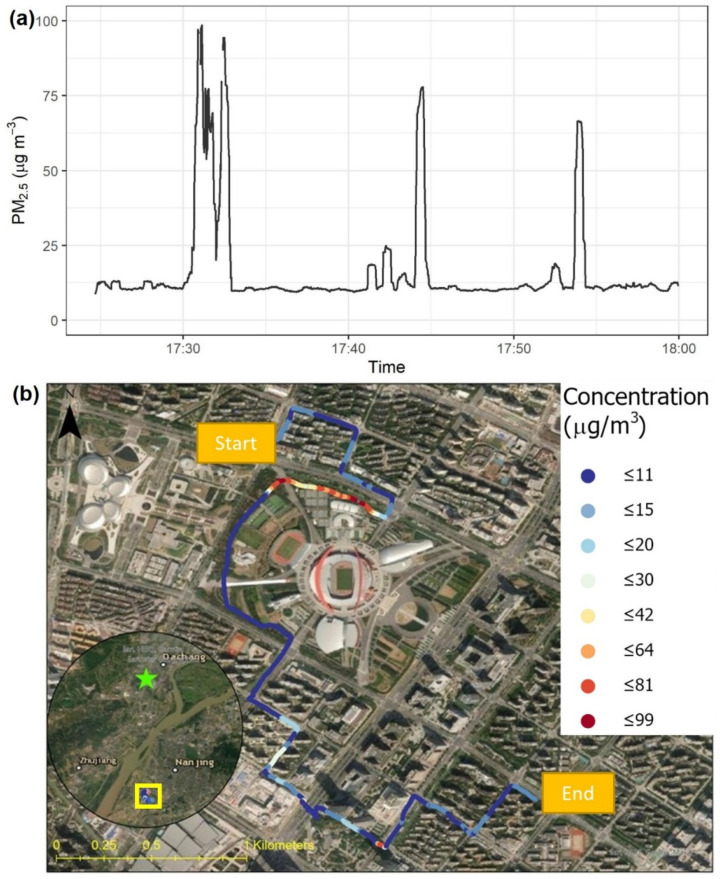
Bicycle-based test in Nanjing, Jiangsu, China using one Smart-P monitor (ID: 508): (**a**) time series of PM_2.5_ concentrations and (**b**) map visualization of PM_2.5_ concentrations. At the time of the test, the hourly average PM_2.5_ concentration from the nearest regulatory monitor (green star) was 41 μg m^−3^.

Figure 10 shows the PM_2.5_ concentration measured with a bicycle riding on sidewalks, designated bicycle lanes, and greenways in Nanjing. For most of this transect, the concentration was relatively stable at about 10 μg m^−3^. This stable background was interrupted by three periods of elevated PM_2.5_ with 7 to 10 times higher concentrations. The first one (peak concentration 99 μg m^−3^) was observed on the greenway north of the Nanjing Olympic Sports Stadium at about 17:31. This greenway was adjacent to the south side of a four-lane arterial road (Mengdu Street), where heavy rush-hour traffic was observed at the time of the measurement. In contrast, no elevated level was measured on the north side of Mengdu Street, indicating the potential role of wind direction in near-road air quality [65]. The other two episodes, at 17:44 and 17:54 with peak concentrations of 78 μg m^−3^ and 66 μg m^−3^, respectively, were observed at traffic intersections, which can be hotspots due to motor vehicle-related emissions [66]. For comparison, the hourly concentration at the nearest regulatory monitoring station was 41 μg m^−3^.

### 3.4. Sensitivity to Travel Speed

**Figure 11:**
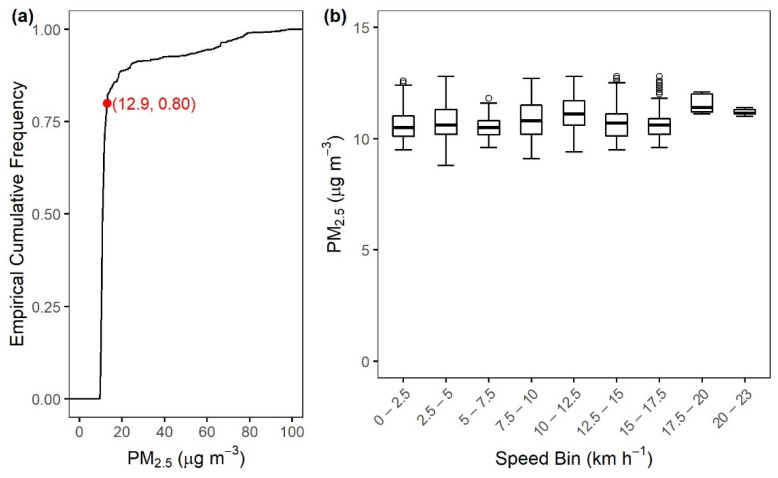
PM_2.5_ concentrations measured by one Smart-P (ID: 508) during the Nanjing bicycle test: (**a**) empirical cumulative distribution and (**b**) relationship between the bottom 80% PM_2.5_ concentrations and cycling speed.

A key difference between mobile and stationary platforms is that mobile platforms involve moving instruments at varying speeds. Travel speed may impact how air enters the sampling tube and instrument chamber, potentially causing artifacts in sensor readings [10,11]. Here we evaluated the measured background PM_2.5_ concentrations vs. speed (Figure 11) for the Nanjing bicycle-based test. In this test, the lowest 80% of the 1 Hz PM_2.5_ concentration data were relatively similar, in the range of 8.8 to 12.9 μg m^−3^ (Figure 11a). We used these 80% of PM_2.5_ concentrations as the basis for the evaluation of the potential impact of speed on Smart-P readings. The top 20% of PM_2.5_ concentrations were excluded for this analysis because they were episodic. The first seven speed bins, from a speed of 0 to 17.5 km h^−1^, had similar concentration ranges (9 to 13 μg m^−3^) and median values (11 μg m^−3^). For these seven speed bins (sample size of one bin = 68 to 498), the Pearson correlation between the bin average concentration and bin average speed was statistically insignificant (*r* = 0.58, *p* = 0.10). The narrower range for speed greater than 17.5 km h^−1^ was caused by a small sample size (≤13 s in one bin).

The insensitivity of the background concentration to speed was also observed during the other mobile measurements (Figures S9–S11). These results suggest that artifacts due to varying speed were negligible.

## 4. Discussion

### 4.1. Measurement Accuracy and Precision

All the Smart-P results were presented based on manufacturer calibration. These results indicated that even with just manufacturer calibration, Smart-P can provide useful information to citizen scientists for applications such as understanding local air quality trends and identifying emission hotspots. Nevertheless, Smart-P accuracy can be further improved by calibration against a high-quality monitor prior to deployment by collocation [44]. The period of collocation should be long enough to cover a wider range of ambient PM_2.5_ concentration, temperature, and RH, and the calibration standard monitor should have the capacity to measure at a high temporal resolution (e.g., 1 min) [34]. Frequent or repetitive collocation strategies are also suggested to better characterize the performance of portable monitors [52]. Ideally, performance characterization should be carried out against a reference monitor on a mobile platform and at fine (e.g., 1 min) temporal resolutions, if portable reference monitors become available in the future. Choice of calibration models matters to the final data quality of low-cost PM sensors [67]. Calibration approaches that use collocated measurements of temperature and RH to correct interferences from temperature and RH on light-scattering PM_2.5_ monitors have been shown to improve performance, with laboratory-calibrated monitors and calibration via collocation using multi-variable gain-offset calibration models increasingly improving RMSE over raw sensor data [45,61,68]. To enable these methods and enhance calibrations, future work can pair a Smart-P monitor with a smart sensor for measuring temperature and RH [32] or modify the Smart-P by adding a temperature and RH sensor to its circuit board.

High precision is necessary to ensure that results from different Smart-P monitors can be merged for mapping spatial patterns when deployed in large quantities. Table S4 compares the precision between stationary and mobile mode. These results covered a wide range of RH, travel speed, and concentration. The precision was in the range of 6% to 13%, which is comparable to a prior test with three SDS011 sensors mounted at a stationary air quality monitoring site [44]. The best precision (6%) was achieved with the bicycle measurement, the primary measurement mode the Smart-P is designed for.

### 4.2. Spatial Variations in PM_2.5_ Concentration

The street tests conducted in three cities confirmed that the Smart-P responded well to capturing large enhancements in PM_2.5_ concentrations, with some contributions from traffic-related air pollution. Multiple PM_2.5_ concentration peaks were measured in each city. These PM_2.5_ peaks were 3 to 50 times higher than the corresponding background concentrations, the latter of which did not appear to be affected by travel speed. The highest peak-to-background ratio was 50 measured in the New Haven car test. The ability to measure these local hotspots is indicative that the Smart-P is ready for monitoring street-level spatial variations in PM_2.5_ concentration.

Bicycles offer two advantages over cars as the measurement platform. First, bicycles have access to places where cars are prohibited. In the Nanjing bicycle test, the highest PM_2.5_ concentration (99 μg m^−3^, about seven times the background concentration) was observed in a greenway despite sheltering in a dense vegetation barrier from street traffic (Figure 10). Data of this kind may help inform how to best shelter bicycle lanes from traffic-related air pollution when they are near roadways [27]. Second, bicycles do not emit pollutants, but tailpipe exhausts from cars can interfere with measurements. Although this problem can be reduced by placing sensors away from the tailpipe of the measurement car, contamination can still occur when the car is stopped (Figure 7).

Our study adds to a growing body of literature that demonstrates the feasibility of and the need for using low-cost sensors on mobile platforms to map spatial variations in PM_2.5_ concentration in the urban environment [33,34,35,36,37,38,39,40,41]. We made two specific contributions. First, we investigated precision, a critical aspect of feasibility, in real-world measurement conditions (varying humidity and travel speed). Second, we conducted measurements in multiple cities with different morphology and background concentrations. In our view, these tests were an important step before the large deployment of low-cost sensors.

A single measurement using the Smart-P, or commercial portable monitors, is not expected to provide high confidence in the concentration at a small spatial scale, such as every 100 m. Repetitive measurement with multiple sensors, such as shown in Figures S5 and S7, or with the same monitor over time along fixed street transects, can allow quantification of statistics, such as mean values and confidence intervals [7,69]. Suggestions on mitigating local air pollution can be made based on these statistics with certain confidence levels. An advantage of Smart-P is that it is designed to facilitate such repetitive measurements.

### 4.3. Potential for Citizen Science

Cost and portability are two barriers to the promotion of citizen science in air quality monitoring. Some commercial monitors are available on the market [57]. Monitors such as AirBeam [41], MicroPEM [40], PocketLab [70], PurpleAir [18], and Speck [71], are portable. However, their costs (USD 250 to 2000 per unit, plus the cost of data-logging and geolocating devices) may still be too high for an average citizen scientist. In comparison, the cost of a Smart-P monitor is only USD 60. We anticipate further cost reduction as cheaper options for Bluetooth, one of the most expensive Smart-P components (Table S1), become available. The enclosure, the other expensive component, was 3D-printed. In mass production, switching from 3D printing to laser cutting or injection molding can reduce the cost of the enclosure by at least 50%.

Bicycles are the primary measurement platform the Smart-P was designed for [20,72]. To improve environmental quality and to promote healthy living, cities across the world are increasingly using bicycles as an eco-friendly, alternative mode of transportation, and motor vehicles remain an important contributor to pollutant emissions in many cities globally [73]. With smart and inexpensive sensors, public and private bicycles have the potential to become a new platform for air monitoring, thus achieving the dual goals of advancing science as well as keeping physical fitness. Towards this end, Smart-P expands the existing choices of low-cost PM monitors.

## 5. Summary

We reported the development of a portable PM_2.5_ monitor called Smart-P for mobile applications. The Smart-P achieved the goals of low-cost (USD 60 per monitor), lightweight (147 g), low power consumption (4 to 105 mA depending on sampling frequency), good precision (6% in mobile mode), ease of use, and smartphone compatibility to facilitate deployment by citizen scientists. The Smart-P used a low-cost optical sensor (SDS011) to measure the PM_2.5_ concentration. Although the performance of the SDS011 in stationary monitoring was documented in the literature, its performance in mobile mode was not known. Bicycle motion may impact how air enters the instrument chamber, potentially producing artifacts in sensor readings. We showed that the measurement was not susceptible to bicycle motion. On-street tests suggest that the measurement precision should be sufficient for mapping spatial variations of PM_2.5_ concentration in urban land with multiple monitors.

The Smart-P allows room for future upgrades. Integration of a low-cost temperature and RH sensor onto the motherboard would facilitate the correction of interferences from temperature and RH on the sensor’s PM detection. Further cost reduction may be achieved by replacing its Bluetooth module with a less expensive one and by switching from 3D printing of the enclosure to laser cutting or injection molding. Another improvement is to power the Smart-P with a small rechargeable battery connected to a bicycle phone charger which harvests the mechanical energy from bicycle motion, thus avoiding the need for a power bank. 

## Figures and Tables

**Table 1:** Summary of linear regression models of hourly average PM_2.5_ concentrations between Smart-P monitors and the BAM-1020 monitor at the Yale Coastal Field Station (YCFS).

Relative Humidity (%)	Dependent Variable	Independent Variable	N *^a^*	Slope	Intercept (µg m^−3^)	*R* ^2^	RMSE *^b^* (µg m^−3^)	MAE *^c^* (µg m^−3^)	MBE *^d^* (µg m^−3^)	BAM-1020 PM_2.5_ (µg m^−3^)		
										Min	Mean	Max
38–100	Smart-P 501 PM_2.5_	BAM-1020 PM_2.5_	167	1.39	−6.22	0.69	10.1	7.1	1.5	2	20	54
38–70			87	1.26	−7.24	0.84	5.8	5.2	−1.9	3	21	54
70–100			80	1.58	−5.78	0.68	11.8	8.9	5.1	5	19	53
38–100	Smart-P 502 PM_2.5_		153	1.31	−7.22	0.72	9.0	6.8	−0.9	3	20	54
38–70			79	1.19	−7.23	0.82	6.0	5.3	−3.2	3	22	54
70–100			74	1.49	−7.63	0.73	10.4	7.3	1.6	5	19	53
38–100	Smart-P 503 PM_2.5_		159	0.89	−1.24	0.53	5.6	5.3	−3.0	5	16	39
38–70			94	0.92	−3.21	0.73	4.3	3.4	−4.4	5	16	39
70–100			65	0.78	2.68	0.25	6.5	5.5	−0.9	8	16	31
38–100	Smart-P 504 PM_2.5_		163	1.37	−6.35	0.74	8.8	6.8	0.7	3	19	54
38–70			79	1.26	−7.50	0.86	5.7	4.7	−2.2	3	20	54
70–100			84	1.53	−6.18	0.73	9.9	7.8	3.4	5	18	53

*^a^* Number of valid hours for comparison. *^b^* Root mean square error. *^c^* Mean absolute error. *^d^* Mean bias error.

**Table 2:** Precision among multiple (3 or 4) Smart-P monitors during collocation measurements at Yale Coastal Field Station (YCFS).

Scenario	Smart-P Monitors	Relative Humidity (%)	# of Hours	SD (µg m^−3^)	CV (%)
1	501, 502, 503, 504	38—100	65	1.6	9
2		38–70	31	0.7	6
3		70–100	34	2.5	12
4	501, 502, 504	38–100	117	1.4	9
5		38–70	54	1.0	6
6		70–100	63	2.2	10

## Data Availability

Data are available from the corresponding author upon request.

## Supplementary Materials

The supporting information can be downloaded at: https://www.mdpi.com/article/10.3390/s22072767/s1.
