# Supplementary material for: Development and Performance Evaluation of a Low-Cost Portable PM2.5 Monitor for Mobile Deployment
Source: Sensors (Basel). 2022 Apr 4;22(7):2767. doi: 10.3390/s22072767 (PMC9003072; doi:10.3390/s22072767)
Supplement: Supplementary file 1 [file sensors-22-02767-s001.zip › sensors-1603392-supplementary.pdf]

Supporting Information for

Development and Performance Evaluation of a Low-Cost Portable $PM_{2.5}$ Monitor for Mobile Deployment

Mingjian Chen [1,2,†], Weichang Yuan [3,*,†], Chang Cao [1,2], Colby Buehler [4,5], Drew R. Gentner [4,5] and Xuhui Lee [3]

[1] Yale-NUIST Center on Atmospheric Environment, Nanjing University of Information Science and Technology, Nanjing 210044, China; 20191207006@nuist.edu.cn (M.C.); chang.cao@nuist.edu.cn (C.C.)

[2] Jiangsu Key Laboratory of Agriculture Meteorology, Nanjing University of Information Science and Technology, Nanjing 210044, China

[3] School of the Environment, Yale University, New Haven, CT 06511, USA; xuhui.lee@yale.edu

[4] Department of Chemical & Environmental Engineering, School of Engineering and Applied Science, Yale University, New Haven, CT 06511, USA; colby.buehler@yale.edu (C.B.); drew.gentner@yale.edu (D.R.G.)

[5] Solutions for Energy, Air, Climate and Health (SEARCH), School of the Environment, Yale University, New Haven, CT 06511, USA

* Correspondence: weichang.yuan@yale.edu

† These authors contributed equally to this work.

Table S1. Breakdown of the costs of the hardware for a Smart-P monitor.

Module	Component	Retail Price (USD) [a]
PM Sensor	SDS011	16.24
Motherboard	Microcontroller: STM32F401RBT6	6.72
	Bluetooth: HC-05	16.24 [b]
	Power Regulator: RT8096CHGJ5	0.31
	Printed Circuit Board (PCB)	7.11
Enclosure	3D Printed parts [c]	10.82

[a] Retail price is based on a Chinese e-commerce website (www.taobao.com).
[b] The current Smart-P prototypes use HC-05 Bluetooth module. Alternatively, HC-02 Bluetooth module ($3.87 USD) can be used to replace HC-05 for cost reduction.
[c] The enclosure was 3D printed at Wenext (https://www.wenext.com/index.php?route=common/home).

Table S2. Overview of the collected data from four Smart-P monitors during the six collocation measurements at the Yale Coastal Field Station (YCFS) in July 2021.

Measurements ID [a]	From		To		Data Availability [b]			
	Date	Time	Date	Time	501	502	503	504
1	7/5/2021	11:37 AM	7/5/2021	6:35 PM	x	x	x	x
2	7/7/2021	10:23 AM	7/8/2021	11:03 AM	x	x	x	
3	7/15/2021	10:14 AM	7/17/2021	2:31 PM	x	x	x	x
4	7/19/2021	10:54 AM	7/21/2021	2:00 PM	x	x		x
5	7/22/2021	10:28 AM	7/24/2021	10:18 AM			x	
6	7/27/2021	10:21 AM	7/28/2021	4:47 PM	x	x	x	x

[a] Measurements were made intermittently due to frequent summer thunderstorms.
[b] Data availability is indicated for each Smart-P monitor (IDs: 501, 502, 503, and 504) using the symbol "x." Data were not collected for a few measurements for one to three Smart-P monitors because of YNCenter APP crashes.

Table S3. Summary of the collected data from four Smart-P monitors during the six collocation measurements at Yale Coastal Field Station (YCFS) in July 2021.

Smart-P ID	n (# of readings) [a]	N (# of valid hours) [b]	Descriptive Statistics of $PM_{2.5}$ Readings (µg m^{-3}) [c]			
			Min	Median	Mean	Max
501	19,664	163	0	17	21	133
502	17,891	146	1	15	19	133
503	18,499	152	2	11	13	51
504	19,126	158	1	16	20	120

[a] For each Smart-P monitor, readings were recorded every 30 seconds.
[b] There are 120 readings from each Smart-P monitor for a given hour that was fully covered during the measurements. Nevertheless, measurement start and end times were typically not at the top of the hour, which led to less than 120 readings per Smart-P monitor for some hours. Thus, we defined a valid hour as the hour with ≥ 90 readings (i.e., 75% of 120).
[c] These descriptive statistics are based on direct readings (one reading every 30 seconds) from each Smart-P monitor.

Table S4. Summary of precision in 1-minute average $PM_{2.5}$ concentrations measured by Smart-P monitors during the tests in Connecticut, U.S.

**Platform Type**	**Test**	**Smart-P ID**	**RH (%)**	**# of Minutes**	**1-minute Average $PM_{2.5}$ (μg m^{-3})**			**SD (μg m^{-3})**	**CV (%)**
					**Min**	**Mean**	**Max**		
Stationary	Collocation at YCFS	501, 502, 503, 504	(38,100]	4,478	2	16	46	1.6	9%
		501, 502, 504		7,535	1	21	126	1.6	8%
	Cooking (Noon)	501, 502, 503, 504	< 70	159	8	48	957	3.8	6%
	Cooking (Evening)			250	5	6	12	0.5	8%
Mobile	New Haven Car			51	2	10	69	1.8	13%
	New Haven Bicycle			31	4	5	8	0.3	6%

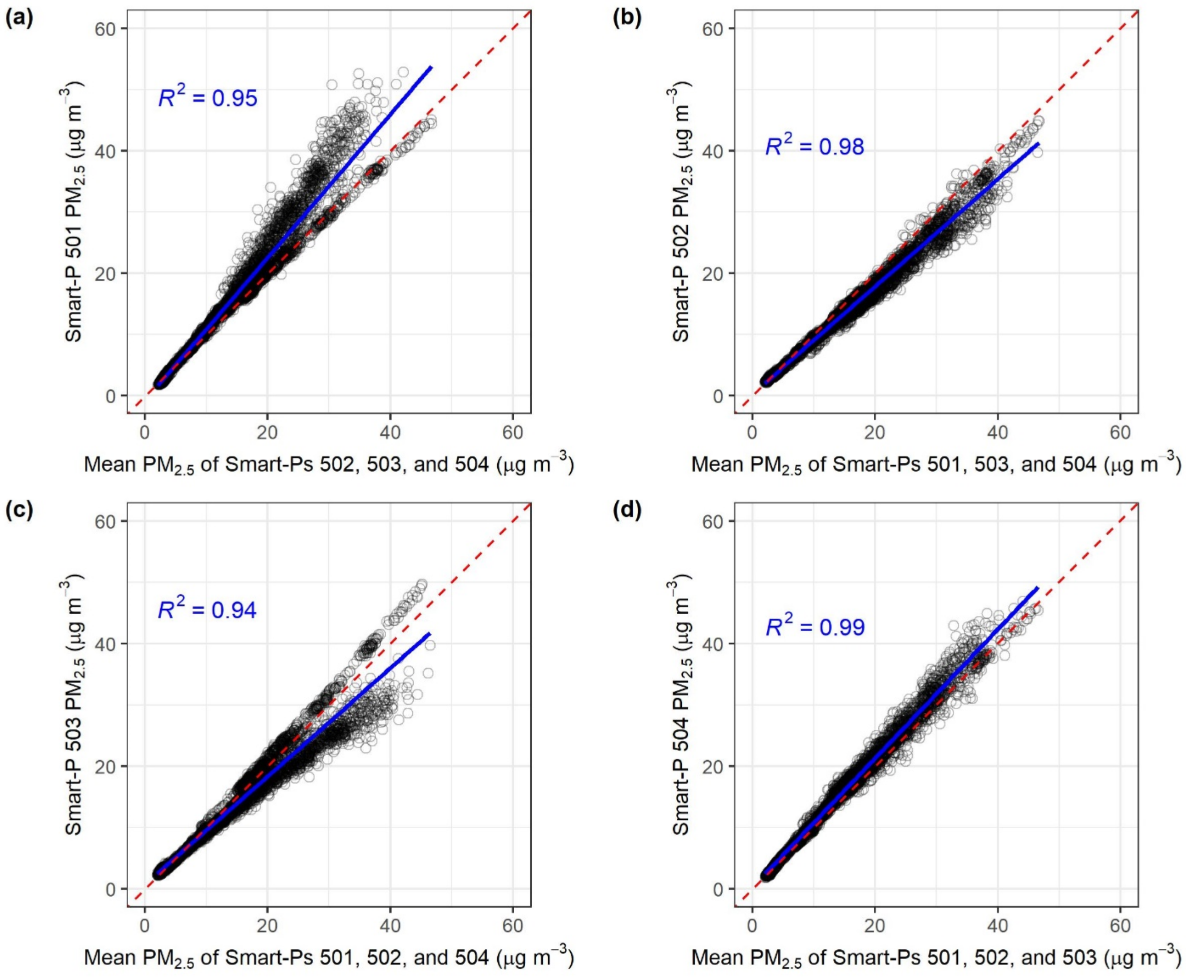

Figure S1. Comparisons of 1-minute average $PM_{2.5}$ concentrations between each of the four Smart-P monitors (IDs: 501, 502, 503, and 504) and the mean value of the other three monitors for the collocation measurements at Yale Coastal Field Station (YCFS): (a) Smart-P 501, (b) Smart-P 502, (c) Smart-P 503, and (d) Smart-P 504. Relative humidity ranged from 38% to 100% for these data. Sample size = 4,478 minutes.

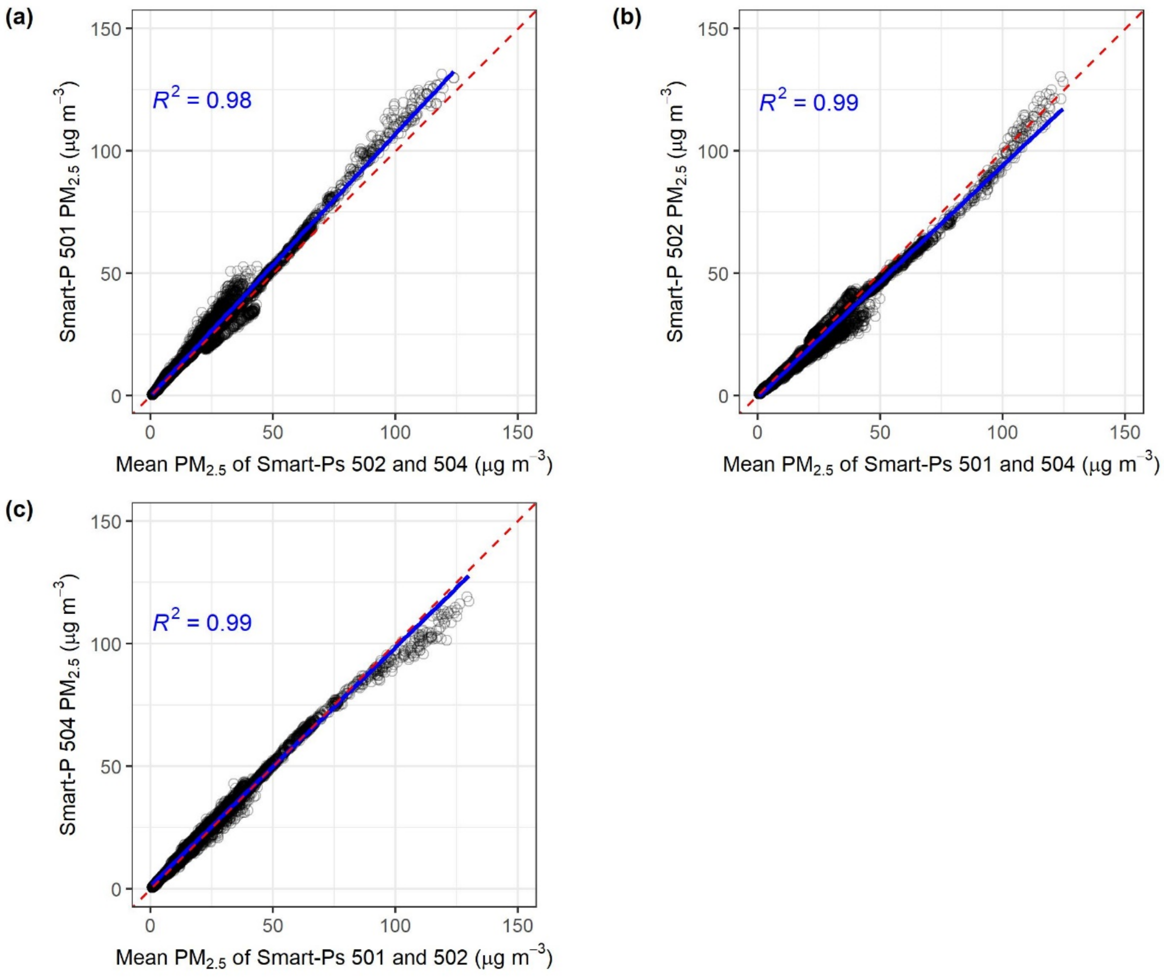

Figure S2. Comparisons of 1-minute average $PM_{2.5}$ concentrations between each of the three Smart-P monitors (IDs: 501, 502, and 504) and the mean value of the other two monitors for the collocation measurements at Yale Coastal Field Station (YCFS): (a) Smart-P 501, (b) Smart-P 502, and (c) Smart-P 504. Relative humidity ranged from 38% to 100% for these data. Sample size = 7,535 minutes.

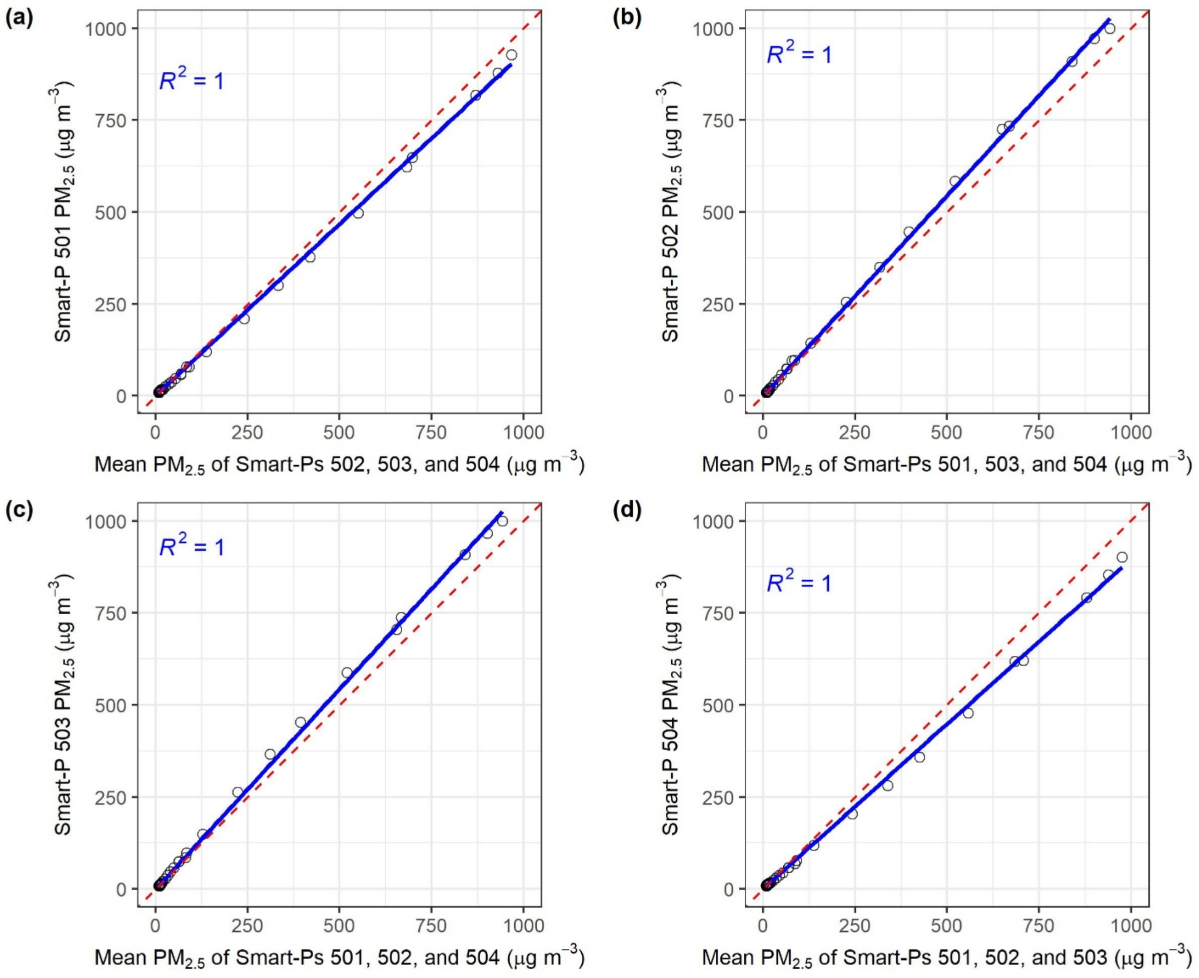

Figure S3. Comparisons of 1-minute average $PM_{2.5}$ concentrations between each of the four Smart-P monitors (IDs: 501, 502, 503, and 504) and the mean value of the other three monitors for the first indoor cooking event with oil during noon time: (a) Smart-P 501, (b) Smart-P 502, (c) Smart-P 503, and (d) Smart-P 504. Sample size = 159 minutes.

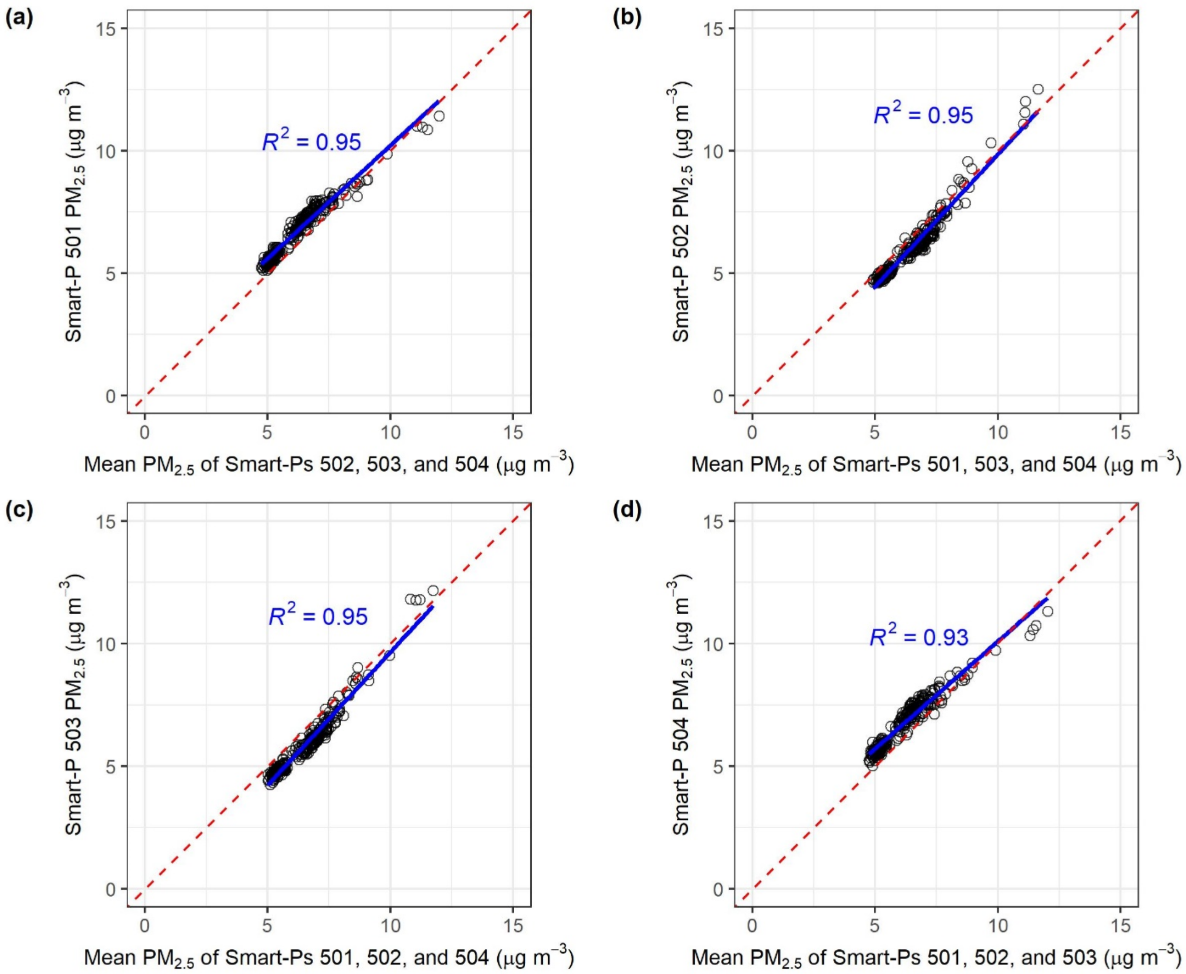

Figure S4. Comparisons of 1-minute average $PM_{2.5}$ concentrations between each of the four Smart-P monitors (IDs: 501, 502, 503, and 504) and the mean value of the other three monitors for the second indoor cooking event with water during evening time: (a) Smart-P 501, (b) Smart-P 502, (c) Smart-P 503, and (d) Smart-P 504. Sample size = 250 minutes.

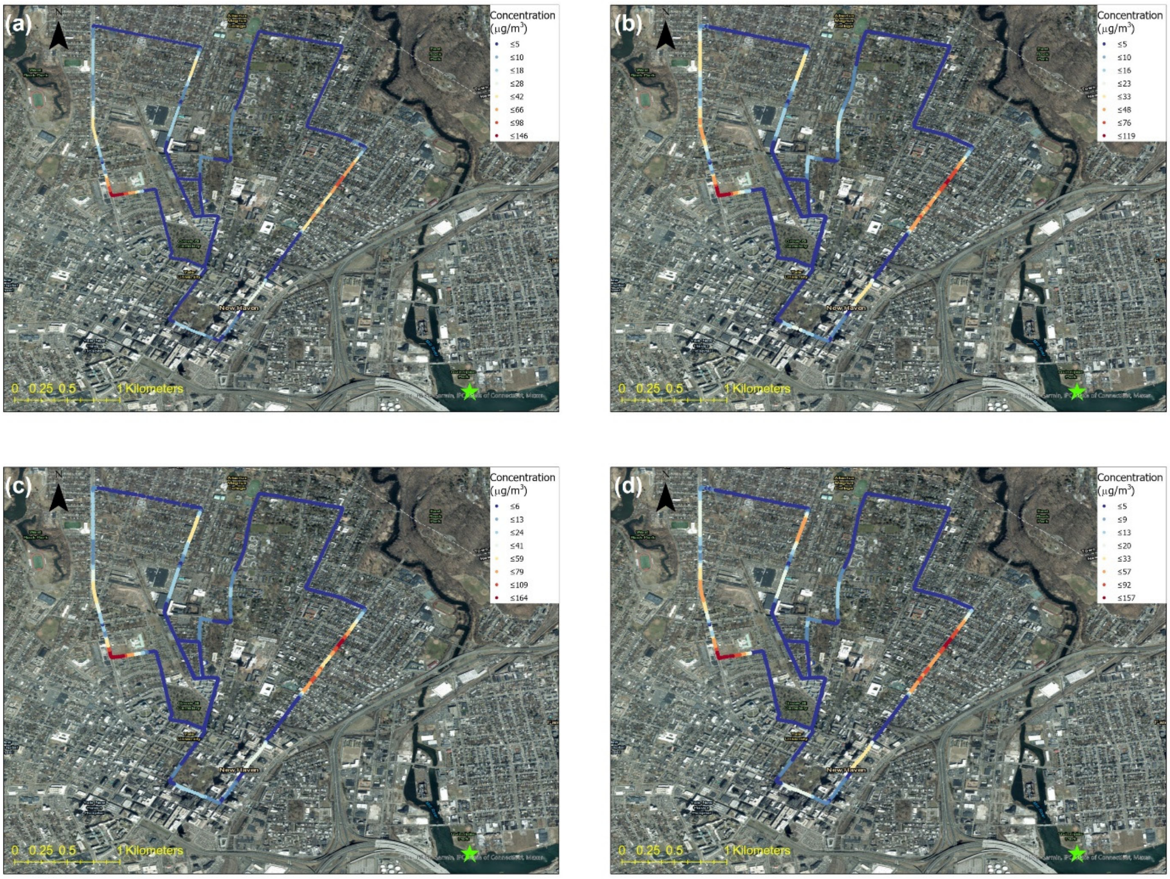

Figure S5. Intra-city variation in $PM_{2.5}$ concentrations measured by four Smart-P monitors during the New Haven car test: (a) Smart-P 501, (b) Smart-P 502, (c) Smart-P 503, and (d) Smart-P 504. At the time of this car test, the hourly-average $PM_{2.5}$ concentration from the nearest regulatory monitor (green star) was 7.5 μg m^{-3}.

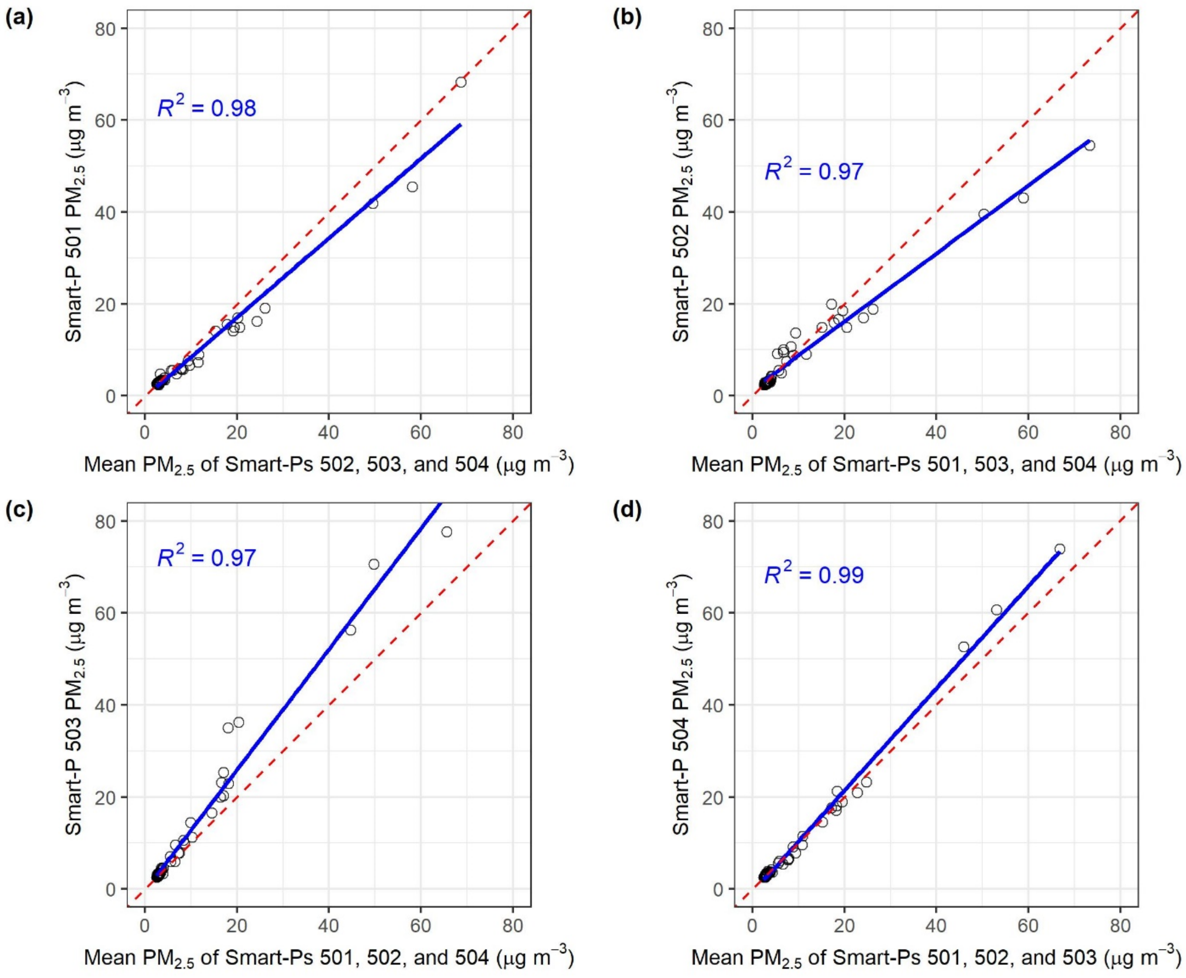

Figure S6. Comparisons of 1-minute average $PM_{2.5}$ concentrations between each of the four Smart-P monitors (IDs: 501, 502, 503, and 504) and the mean value of the other three monitors for the car test in New Haven, Connecticut, U.S.: (a) Smart-P 501, (b) Smart-P 502, (c) Smart-P 503, and (d) Smart-P 504. Sample size = 51 minutes.

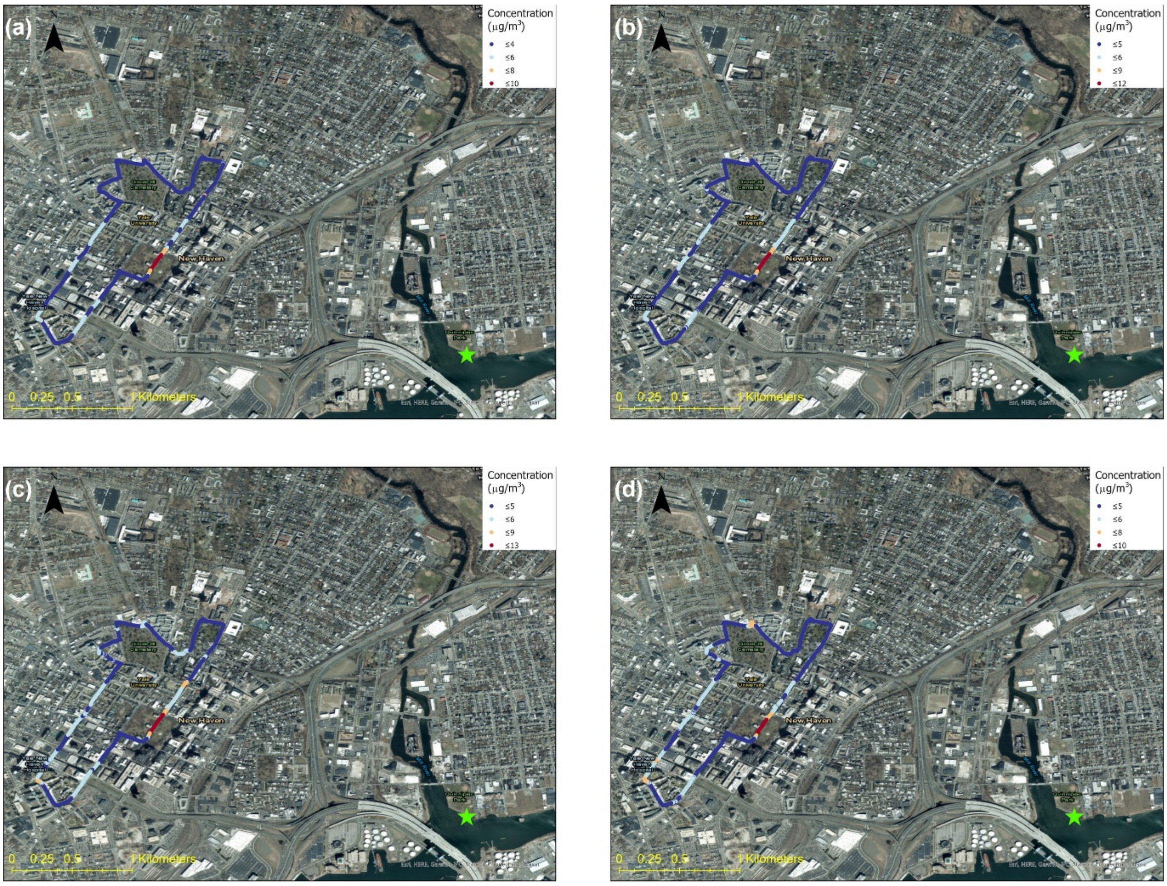

Figure S7. Intra-city variation in $PM_{2.5}$ concentrations measured by four Smart-P monitors during the New Haven bicycle test: (a) Smart-P 501, (b) Smart-P 502, (c) Smart-P 503, and (d) Smart-P 504. At the time of this bicycle test, the hourly-average $PM_{2.5}$ concentration from the nearest regulatory monitor (green star) was 8.5 μg m^{-3}.

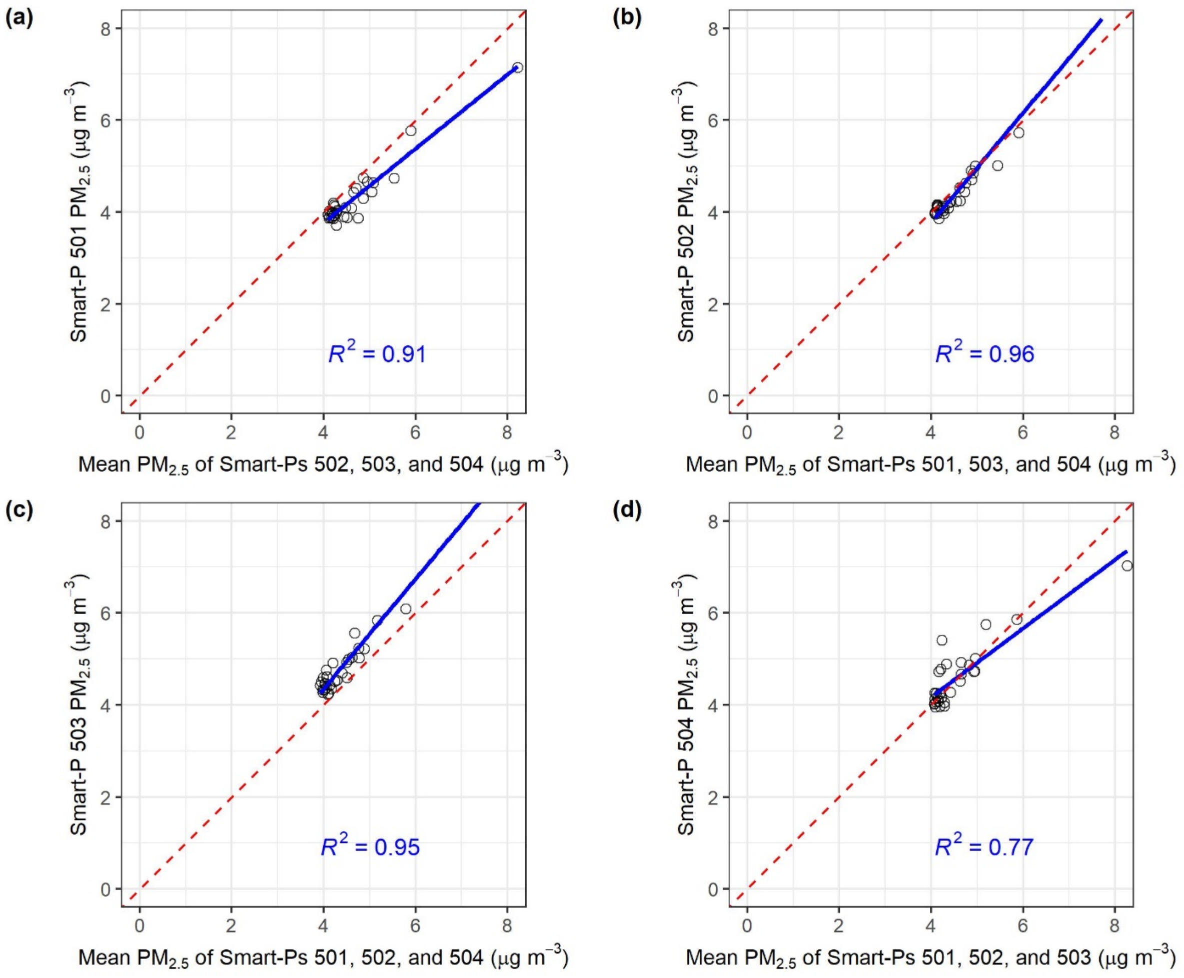

Figure S8. Comparisons of 1-minute average $PM_{2.5}$ concentrations between each of the four Smart-P monitors (IDs: 501, 502, 503, and 504) and the mean value of the other three monitors for the bicycle test in New Haven, Connecticut, U.S.: (a) Smart-P 501, (b) Smart-P 502, (c) Smart-P 503, and (d) Smart-P 504. Sample size = 31 minutes.

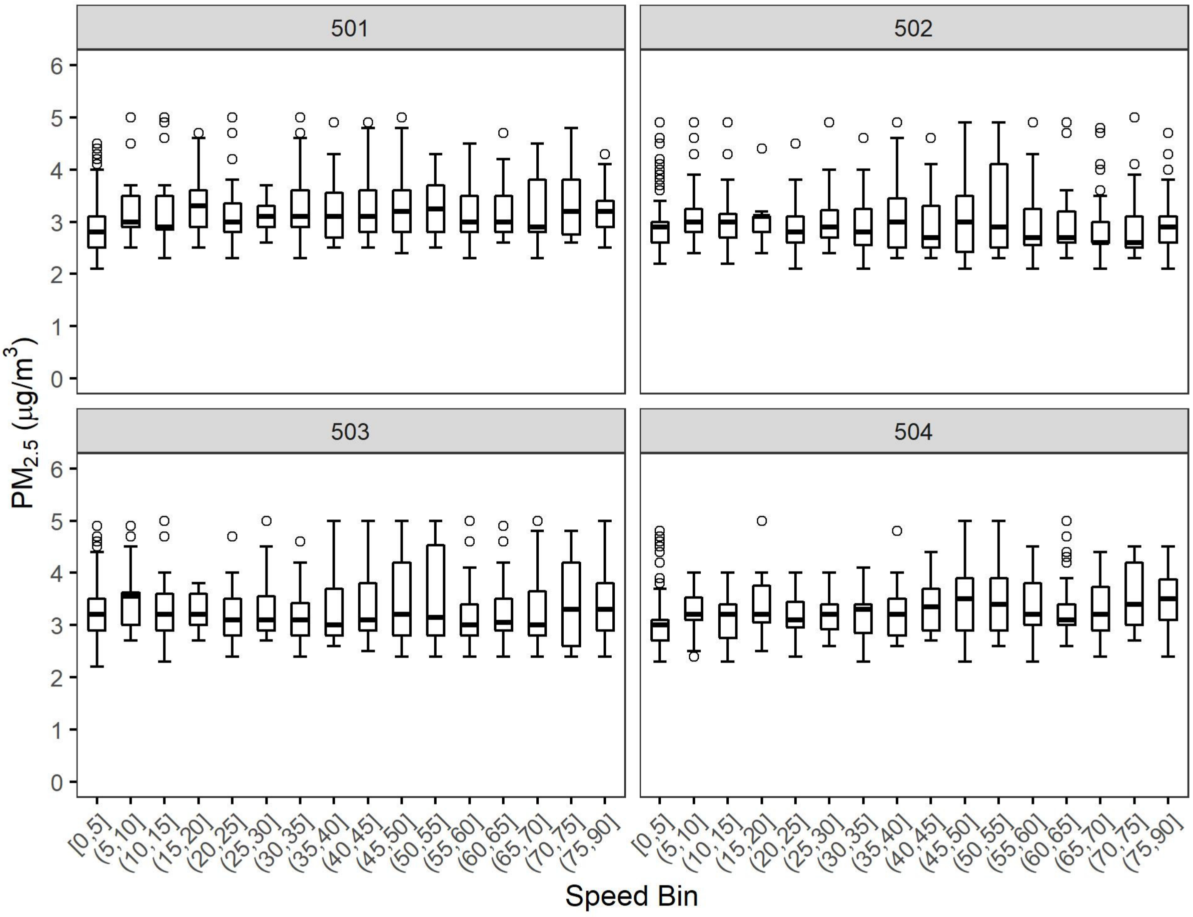

Figure S9. Relationship between background $PM_{2.5}$ concentrations measured by four Smart-P monitors (IDs: 501, 502, 503, and 504) and driving speed during the New Haven car test.

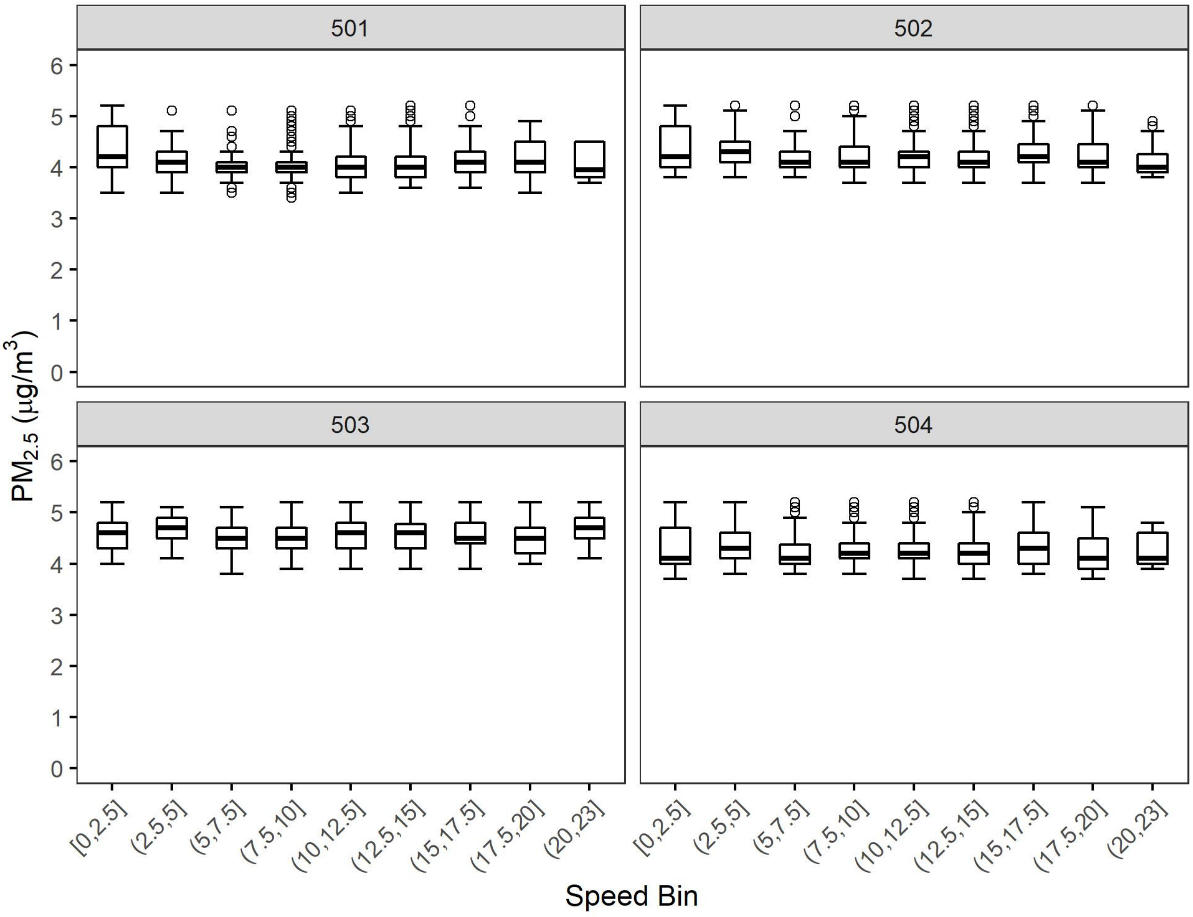

Figure S10. Relationship between background $PM_{2.5}$ concentrations measured by four Smart-P monitors (IDs: 501, 502, 503, and 504) and cycling speed during the New Haven bicycle test.

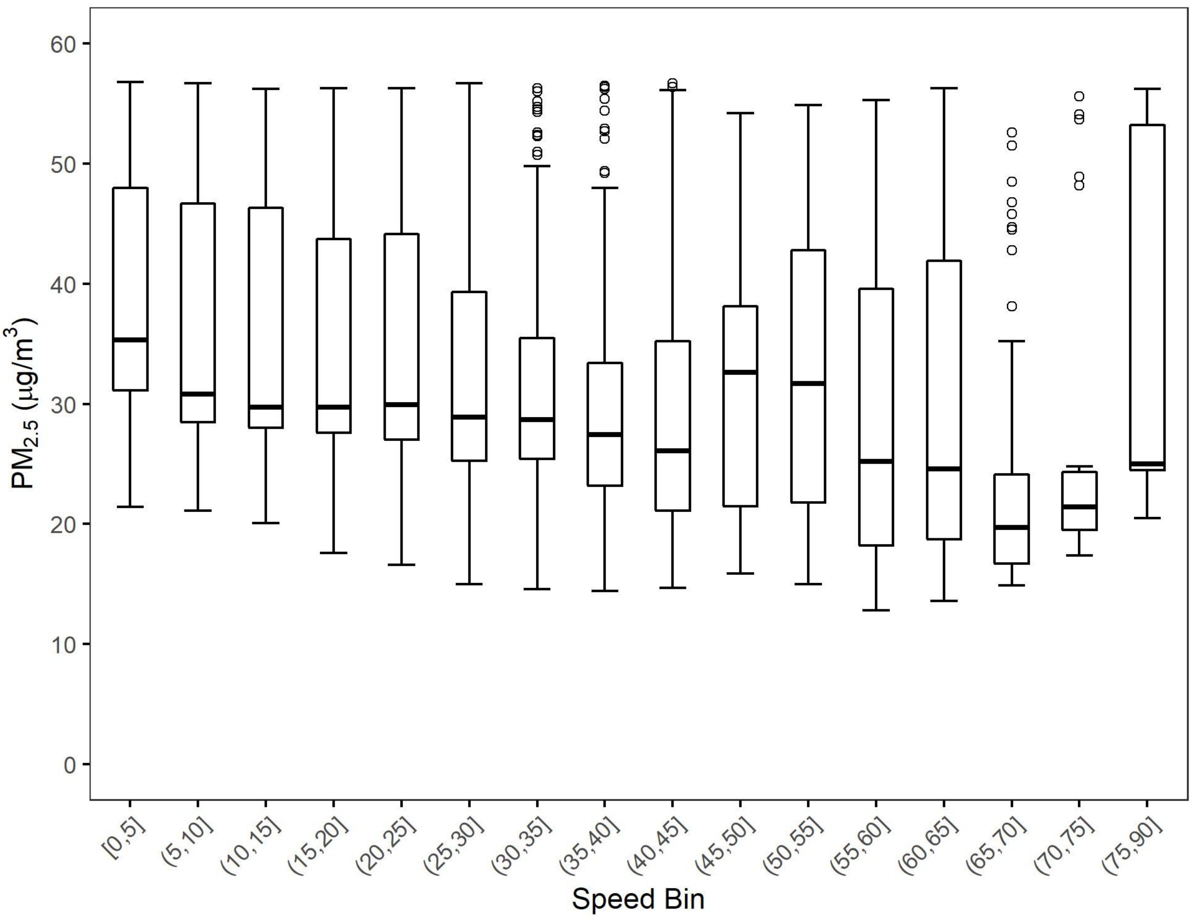

Figure S11. Relationship between $PM_{2.5}$ concentrations measured by one Smart-P monitor (ID: 508) and driving speed during the Hangzhou car test.
